# The Topographical, Sensory, and Temporal Characteristics of Pain in Parkinson’s Disease: A Cross‐Sectional Survey

**DOI:** 10.1111/papr.70142

**Published:** 2026-03-11

**Authors:** Anthony Mezzini, Saravana Kumar, Sue Sharrad, Joanne Harmon, Marion Eckert

**Affiliations:** ^1^ Rosemary Bryant AO Research Centre, College of Health Adelaide University Adelaide South Australia Australia; ^2^ School of Allied Health and Human Performance, College of Health Adelaide University Adelaide South Australia Australia

**Keywords:** characteristics, pain, Parkinson’s disease, sensory, temporal, topography

## Abstract

**Background:**

Pain in Parkinson's disease (PD) manifests clinically in a variety of ways and several pain types have been identified, however, their characteristics have not been extensively studied.

**Objective:**

To describe the characteristics of common pain types in people with PD.

**Methods:**

A survey of people with PD that experience pain symptoms was conducted. Participants were recruited using a combination of sampling methods. The survey questions focused on collecting information about the topographical, sensory, and temporal characteristics of pain. Descriptive statistics were used to report results.

**Results:**

Two hundred and sixteen participants were included in the data analyses. A median of 5 (interquartile range [IQR] 4–7) pain types were experienced by participants. Pain was commonly localized in the lower limbs but often affected several different body segments. Acute attacks of pain were characteristic for many participants in most pain types and were typically frequent (two to six times per week or more) and prolonged (minutes to hours). Periods of pain accentuation were most likely to occur in the morning, evening, and night and appear to be associated with PD medication pharmacology and a variety of exacerbating and ameliorating factors. Fluctuations in pain were found to range from mild to severe in all pain types.

**Conclusions:**

This study makes a valuable contribution to the existing literature by providing the most detailed assessment of the topographical, sensory, and temporal characteristics of common PD pain types to date that may be used to guide clinical diagnosis and treatment.

## 1 Introduction

Pain is among the most prevalent and debilitating non‐motor symptom of Parkinson's disease (PD) [1, 2, 3, 4, 5, 6]. Increasing knowledge of PD pain pathomechanisms has found that disease specific neurodegenerative changes occur in the cerebral, spinal, and peripheral nociceptive pathways, and involves deficits in dopaminergic and nondopaminergic neurotransmitter systems as well as variations in several genes involved in the processing of nociceptive inputs [7]. These pathophysiological changes may interact with comorbidities associated with painful symptoms (i.e., osteoarthritis) and PD motor symptoms (i.e., bradykinesia and rigidity), and as a result, the clinical manifestations of PD pain vary greatly [8, 9].

Several systems for categorizing the variety of PD pain symptoms into pain types have been proposed in the literature [9, 10, 11, 12, 13, 14, 15, 16], and the topographical, and sensory and affective qualities (i.e., intensity and unpleasantness) have been well documented [15, 17, 18, 19, 20, 21]. Few studies, however, have investigated the temporal characteristics (i.e., duration, variability, and modifying factors) of PD pain that are argued to represent distinct and important dimensions of pain for the development of effective treatment approaches [22, 23], or differentiated between the different pain types when investigating and describing pain characteristics. The aim of this study is to address this knowledge gap by describing the characteristics of common pain types in people with PD.

## 2 Methods

### 2.1 Design

This was a cross‐sectional study using an online survey. The study protocol was approved by the University of South Australia's Human Research Ethics Committee (ID: 205671) and reported following the Strengthening the Reporting of Observational Studies in Epidemiology (STROBE) guidelines [24]. The methods follow the approach outlined by Mezzini et al. [25] (currently under peer review) and the methods description herein partly reproduces their wording.

### 2.2 Sample and Recruitment

People with PD living in Australia that experience pain were targeted for this study. Based on an estimated target population of 66,589 [21, 26], a sample size of 382 participants was required to achieve a ±5% margin of error and 95% confidence interval for each question on the survey. A sample size of 150 or more, however, was considered sufficient given the sample size of previous similar studies [17, 18, 27]. Eligibility criteria included: (a) a diagnosis of PD by a neurologist or movement disorder specialist; (b) self‐reported pain symptoms in the past month; (c) Australian citizenship or permanent residency and currently living in Australia; and (d) aged 18 years or older.

There is no suitable sampling frame or central repository for the target population in Australia, and therefore, a combination of self‐selection, network, and purposive non‐probability sampling methods were used to recruit participants [28]. To reach potential participants, the survey was advertised through several PD advocacy and research funding bodies (i.e., The Hospital Research Foundation Group—Parkinson's, Shake It Up Foundation, Parkinson's NSW, QLD and WA, and Fight Parkinson's), PD research and treatment clinics/centers (i.e., The University of Sydney's Brain and Mind Centre, and Advance Rehab Centre), and the clinical and professional networks of the research team via websites, newsletters, social media, email, and brochures.

### 2.3 Measurement

#### 2.3.1 Survey Development

A customized survey instrument was developed by the corresponding author in consultation with the co‐authors and other subject matter experts (i.e., PD nurse specialists, people with PD, and academics with expertise in survey development) following best practice guidelines [29, 30]. Question pathways were individualized, using skip logic branching, depending on participant responses. Survey questions were closed‐ended with either nominal, ordinal or numerical response formats. The draft survey instrument was pilot tested in a non‐probability purposive sample of 10 participants that met the study's eligibility criteria. Participants were asked to identify areas of the survey that caused confusion (i.e., poorly worded questions and survey instructions), to record completion time, and to assess the clinical sensibility (i.e., comprehensiveness, salience, acceptability, and face and content validity) of the instrument. Based on the feedback obtained, clearer and richer descriptions of pain types were provided in the survey.

#### 2.3.2 Survey Questions

In addition to questions collecting basic demographic (age, sex, and residential postcode) and clinical (disease stage, duration, and primary treating medical practitioner) information, the survey comprised three major sections. Section (A) focused on collecting information about the characteristics of PD pain types, Section (B) on the reporting of pain to health care professionals, and Section (C) on pain treatment practices. The results from Sections (B) and (C) will be reported in another study, and therefore are not discussed further.

For Section (A) a novel classification system for categorizing PD pain into pain types was developed for this study, the aim of which was to have pain types with specific characteristics that could be applied in a self‐administered survey. To ensure a strong evidence base, we used several features from previous classification systems [10, 11, 14] along with recent epidemiological PD pain research [31, 32, 33, 34], to develop a system that was composed of 10 pain types: dystonic, dyskinetic, temporomandibular, musculoskeletal, abdominal visceral, radicular neuropathic, peripheral neuropathic, restless legs syndrome, nocturnal akinesia, and central Parkinson's pain. Categories of pain types were not created for syndromes, where, in people with PD, there is insufficient evidence to suggest an elevated risk compared to the general population, are associated with unpleasant sensations rather than pain, or are drug induced. An exception to the latter criteria was made for dyskinesia on account that it does not simply stem from dopamine replacement therapy (levodopa) but also involves PD driven neurological pathophysiology [35], and therefore, can be considered a feature of the disease itself. To minimize misinterpretation and overlap between pain types, detailed descriptions for each category were developed based on previous reports [10, 31, 36, 37, 38, 39, 40, 41, 42], and instructions were carefully worded to reduce the likelihood of participants reporting the same symptom in multiple categories. In addition, the order of pain types was deliberately structured to further minimize confusion and avoid double counting (e.g., temporomandibular pain was presented before musculoskeletal pain).

For each pain type, participants were asked whether they have experienced the described symptoms in the past month (yes/no). If they responded ‘no’, participants were branched to the next pain type. If ‘yes’, participants were then presented with several questions characterizing the pain in terms of its duration, topography, temporal pattern, time and PD medication dependent accentuation, exacerbating and ameliorating factors, and intensity. These questions were either drawn from previous pain questionnaires [43, 44, 45, 46] and modified, where appropriate, for the purposes of this study or self‐developed. Questions stems and responses were kept consistent for each of the pain types, however, exceptions were made where appropriate (i.e., question responses relating to topography were different for musculoskeletal pain as compared to restless legs syndrome [RLS]). A copy of the survey instrument is provided in Material S1.

### 2.4 Data Collection

The survey was administered using the online Qualtrics^XM^ platform. Interested participants that accessed the survey were required to provide informed consent and complete an eligibility criteria checklist. Participants were authorized to complete the survey in stages and be physically assisted should that be their preference. Data were collected between November 2023 and April 2024.

### 2.5 Data Analyses

Survey data were exported to SPSS (version 29) for analyses. Data were first inspected for erroneous responses (i.e., straightlining, speeders, bots), incomplete responses, and missing data. Data sets where Section (A) was incomplete, contained missing data of more than 5%, or were identified as erroneous were excluded from further analyses. Descriptive statistics for categorial data were reported using frequency counts and percentages, and for numerical data, medians and interquartile ranges as the data were not normally distributed.

## 3 Results

Two hundred and seventy‐three eligible participants responded to the survey and 220 reached the end of Section (A). Twenty‐one of the completed data sets (9.5%) contained one or more missing answers and three of these had a percentage of unanswered questions that exceeded 5% (only questions that participants saw are included in this calculation). One erroneous response was also found in the data. A total of 216 participants were included in the results for this study.

### 3.1 Participant Demographics and Clinical Characteristics

The median age of participants was 70 years (interquartile range [IQR] 63–76) and 51.9% were female (Table 1). Most participants were from South Australia (30.1%), New South Wales (22.7%), Victoria (19.0%), and Queensland (14.8%). Almost one‐half of participants (47.7%) had been diagnosed with PD less than 6 years ago and the majority (87.5%) identified with symptoms consistent with stages 1, 2, and 3 of the Hoehn and Yahr 5‐point staging scale [47]. 82.9% of participants indicated that for the treatment of PD a neurologist was their primary treating medical practitioner.

**TABLE 1:** Participant demographic and clinical characteristics (*N* = 216).

Characteristics	Values (*N* = 216), *n* [%]
*Sex*	
Female	112 [51.9]
Male	103 [47.7]
Other	1 [0.5]
Prefer not to say	0 [0.0]
*Age*	
Years (median [IQR])	70 [63–76]
*Current residential location by state and territory*	
New South Wales	49 [22.7]
Australian Capital Territory	9 [4.2]
Victoria	41 [19.0]
Queensland	32 [14.8]
South Australia	65 [30.1]
Western Australia	8 [3.7]
Tasmania	11 [5.1]
Northern Territory	1 [0.5]
*Disease duration* ^a^	
Less than 6 years	103 [47.7]
6 years or more	113 [52.3]
*Disease stage* ^b^	
Stage 1	68 [31.5]
Stage 2	55 [25.5]
Stage 3	66 [30.6]
Stage 4	22 [10.2]
Stage 5	5 [2.3]
*Primary treating medical practitioner*	
Neurologist	179 [82.9]
Geriatrician	14 [6.5]
General practitioner	17 [7.9]
Other	6 [2.8]

^a^ Estimated by subtracting the year of Parkinson's diagnosis provided by participants from 2024.

^b^ Based on the Hoehn and Yahr 5‐point Parkinson's staging scale.

### 3.2 Characteristics of Pain Types

The most frequently reported pain types by participants were musculoskeletal (94.0%) and dystonic (67.6%), and the least, central Parkinson's (34.7%) and temporomandibular (23.1%). A median of 5 (IQR 4–7) pain types were experienced by participants. A very large proportion of participants reported pain as being present for 3 months or more (from 83.7% for temporomandibular pain to 98.0% for musculoskeletal pain), and most for more than 1 year (from 53.1% for temporomandibular pain to 84.4% for RLS).

#### 3.2.1 Temporal Pattern, Time and PD Medication Dependent Accentuation, and Exacerbating and Ameliorating Factors

For pain types where the temporal pattern of pain was investigated (nocturnal akinesia was excluded), many participants reported acute attacks of pain with either pain‐free episodes in between or on a background of constant pain (from [99/203, 48.8%] for musculoskeletal pain to [79/108, 73.1%] for abdominal visceral pain) (Table 2). A high proportion of these acute pain attacks occurred two to six times per week or more (from [15/27, 55.6%] for temporomandibular pain to [90/99, 90.9%] for musculoskeletal pain) and were present for minutes to hours (from [16/27, 59.3%] for temporomandibular pain to [56/61, 91.8%] for RLS).

**TABLE 2:** Temporal pattern, time and PD medication dependent accentuation, and exacerbating and ameliorating factors.

Question stems and responses	Values (*n* [%])									
	Dystonic	Dyskinetic	Temporomandibular	Musculoskeletal	Abdominal Visceral	Radicular Neuropathic	Peripheral Neuropathic	Restless Legs Syndrome	Nocturnal Akinesia	Central Parkinson's
*Pain in the past month*										
Yes	146/216 [67.6]	90/216 [41.7]	50/216 [23.1]	203/216 [94.0]	108/216 [50.0]	123/216 [56.9]	106/216 [49.1]	96/216 [44.4]	145/216 [67.1]	75/216 [34.7]
No	70/216 [32.4]	126/216 [58.3]	166/216 [76.9]	13/216 [6.0]	108/216 [50.0]	93/216 [43.1]	110/216 [50.9]	120/216 [55.6]	71/216 [32.9]	141/216 [65.3]
*Duration*										
Less than 3 months	4/146 [2.7]	5/90 [5.6]	8/49 [16.3]	4/202 [2.0]	10/107 [9.3]	7/123 [5.7]	3/106 [2.8]	4/96 [4.2]	4/145 [2.8]	4/75 [5.3]
3 months to less than 1 year	40/146 [27.4]	22/90 [24.4]	15/49 [30.6]	37/202 [18.3]	32/107 [29.9]	32/123 [26.0]	28/106 [26.4]	11/96 [11.5]	27/145 [18.6]	24/75 [32.0]
More than 1 year	102/146 [69.9]	63/90 [70.0]	26/49 [53.1]	161/202 [79.7]	65/107 [60.7]	84/123 [68.3]	75/106 [70.8]	81/96 [84.4]	114/145 [78.6]	47/75 [62.7]
*Frequency*										
Every night	n/a	n/a	n/a	n/a	n/a	n/a	n/a	n/a	102/144 [70.8]	n/a
Two to six times a week	n/a	n/a	n/a	n/a	n/a	n/a	n/a	n/a	30/144 [20.8]	n/a
Once a week	n/a	n/a	n/a	n/a	n/a	n/a	n/a	n/a	7/144 [4.9]	n/a
Less than once a week	n/a	n/a	n/a	n/a	n/a	n/a	n/a	n/a	5/144 [3.5]	n/a
*Temporal pattern*										
Continuous pain with slight variations	28/145 [19.3]	25/89 [28.1]	19/49 [38.8]	69/203 [34.0]	20/108 [18.5]	33/123 [26.8]	34/105 [32.4]	26/95 [27.4]	n/a	30/74 [40.5]
Continuous pain with large variations	15/145 [10.3]	8/89 [9.0]	3/49 [6.1]	35/203 [17.2]	9/108 [8.3]	12/123 [9.8]	8/105 [7.6]	9/95 [9.5]	n/a	4/74 [5.4]
Acute attacks of pain with pain free periods in‐between	83/145 [57.2]	42/89 [47.2]	26/49 [53.1]	70/203 [34.5]	74/108 [68.5]	68/123 [55.3]	59/105 [56.2]	56/95 [58.9]	n/a	35/74 [47.3]
Acute attacks of pain on a background of constant pain	19/145 [13.1]	14/89 [15.7]	1/49 [2.0]	29/203 [14.3]	5/108 [4.6]	10/123 [8.1]	4/105 [3.8]	4/95 [4.2]	n/a	5/74 [6.8]
*Frequency of acute pain attacks*										
Several times a day	30/103 [29.1]	16/57 [28.1]	4/27 [14.8]	50/99 [50.5]	7/79 [8.9]	23/78 [29.5]	16/64 [25.0]	10/61 [16.4]	n/a	9/40 [22.5]
Once a day	15/103 [14.6]	11/57 [19.3]	3/27 [11.1]	16/99 [16.2]	9/79 [11.4]	6/78 [7.7]	12/64 [18.8]	9/61 [14.8]	n/a	2/40 [5.0]
Two to six times a week	37/103 [35.9]	21/57 [36.8]	8/27 [29.6]	24/99 [24.2]	34/79 [43.0]	27/78 [34.6]	26/64 [40.6]	22/61 [36.1]	n/a	12/40 [30.0]
Once a week	11/103 [10.7]	5/57 [8.8]	5/27 [18.5]	4/99 [4.0]	11/79 [13.9]	11/78 [14.1]	8/64 [12.5]	9/61 [14.8]	n/a	10/40 [25.0]
Less than once a week	10/103 [9.7]	4/57 [7.0]	7/27 [25.9]	5/99 [5.1]	18/79 [22.8]	11/78 [14.1]	2/64 [3.1]	11/61 [18.0]	n/a	7/40 [17.5]
*Duration of acute pain attacks*										
Seconds	21/103 [20.4]	8/57 [14.0]	9/27 [33.9]	5/99 [5.1]	5/79 [6.3]	8/78 [10.3]	8/64 [12.5]	3/61 [4.9]	n/a	5/41 [12.2]
Minutes	49/103 [47.6]	28/57 [49.1]	11/27 [40.7]	39/99 [39.4]	50/79 [63.3]	36/78 [46.2]	34/64 [53.1]	31/61 [50.8]	n/a	20/41 [48.8]
Hours	32/103 [31.1]	20/57 [35.1]	5/27 [18.5]	47/99 [47.5]	21/79 [26.6]	33/78 [42.3]	21/64 [32.8]	25/61 [41.0]	n/a	15/41 [36.6]
Days	1/103 [1.0]	1/57 [1.8]	2/27 [7.4]	8/99 [8.1]	3/79 [3.8]	1/78 [1.3]	1/64 [1.6]	2/61 [3.3]	n/a	1/41 [2.4]
*Time‐dependent accentuation of pain (day/night occurring pain)* ^a^										
Morning	42/146 [28.8]	17/90 [18.9]	10/50 [20.0]	76/203 [37.4]	27/108 [25.0]	29/123 [23.6]	20/106 [18.9]	10/96 [10.4]	n/a	15/75 [20.0]
Noon	3/146 [2.1]	3/90 [3.3]	1/50 [2.0]	12/203 [5.9]	6/108 [5.6]	5/123 [4.1]	1/106 [0.9]	0/96 [0.0]	n/a	2/75 [2.7]
Afternoon	24/146 [16.4]	15/90 [16.7]	7/50 [14.0]	41/203 [20.2]	15/108 [13.9]	18/123 [14.6]	9/106 [8.5]	14/96 [14.6]	n/a	13/75 [17.3]
Evening	46/146 [31.5]	35/90 [38.9]	12/50 [24.0]	63/203 [31.0]	29/108 [26.9]	34/123 [27.6]	32/106 [30.2]	50/96 [52.1]	n/a	25/75 [33.3]
Night	57/146 [39.0]	32/90 [35.6]	13/50 [26.0]	56/203 [27.6]	19/108 [17.6]	34/123 [27.6]	35/106 [33.0]	70/96 [72.9]	n/a	17/75 [22.7]
No difference	35/146 [24.0]	29/90 [32.2]	23/50 [46.0]	77/203 [37.9]	52/108 [48.1]	54/123 [43.9]	45/106 [42.5]	11/96 [11.5]	n/a	39/75 [52.0]
*Time‐dependent accentuation of pain (night occurring pain)* ^a^										
In the first third of the night	n/a	n/a	n/a	n/a	n/a	n/a	n/a	n/a	34/145 [23.4]	n/a
In the middle of the night	n/a	n/a	n/a	n/a	n/a	n/a	n/a	n/a	32/145 [22.1]	n/a
In the last third of the night	n/a	n/a	n/a	n/a	n/a	n/a	n/a	n/a	42/145 [29.0]	n/a
No difference	n/a	n/a	n/a	n/a	n/a	n/a	n/a	n/a	58/145 [40.0]	n/a
*PD medication‐dependent accentuation of pain* ^a^										
Peak‐dose periods	9/146 [6.2]	15/90 [16.7]	4/50 [8.0]	7/203 [3.4]	4/108 [3.7]	1/123 [0.8]	4/106 [3.8]	4/96 [4.2]	1/145 [0.7]	3/75 [4.0]
Wearing‐off or off periods	79/146 [54.1]	36/90 [40.0]	23/50 [46.0]	70/203 [34.5]	13/108 [12.0]	34/123 [27.6]	33/106 [31.1]	38/96 [39.6]	54/145 [37.2]	19/75 [25.3]
Early‐dose periods	11/146 [7.5]	13/90 [14.4]	3/50 [6.0]	10/203 [4.9]	4/108 [3.7]	11/123 [8.9]	7/106 [6.6]	8/96 [8.3]	3/145 [2.1]	5/75 [6.7]
No difference	56/146 [38.4]	36/90 [40.0]	19/50 [38.0]	117/203 [57.6]	81/108 [75.0]	80/123 [65.0]	65/106 [61.3]	53/96 [55.2]	81/145 [55.9]	54/75 [72.0]
Don't take Parkinson's medication	7/146 [4.8]	1/90 [1.1]	4/40 [8.0]	9/203 [4.4]	7/108 [6.5]	5/123 [4.1]	7/106 [6.6]	4/96 [4.2]	7/145 [4.8]	3/75 [4.0]
*Exacerbating factors* ^a^										
Nothing/not sure	25/146 [17.1]	30/90 [33.3]	17/50 [34.0]	37/202 [18.3]	34/108 [31.5]	48/123 [39.0]	49/106 [46.2]	50/96 [52.1]	79/143 [55.2]	39/75 [52.0]
Eating	7/146 [4.8]	4/90 [4.4]	6/50 [12.0]	1/202 [0.5]	41/108 [38.0]	5/123 [4.1]	1/106 [0.9]	1/96 [1.0]	1/143 [0.7]	7/75 [9.3]
Speaking	8/146 [5.5]	10/90 [11.1]	7/50 [14.0]	7/202 [3.2]	2/108 [1.9]	2/123 [1.6]	0/106 [0.0]	0/96 [0.0]	1/143 [0.7]	4/75 [5.3]
Hot weather	25/146 [17.1]	12/90 [13.3]	2/50 [4.0]	27/202 [13.4]	6/108 [5.6]	11/123 [8.9]	8/106 [7.5]	9/96 [9.4]	12/143 [8.4]	12/75 [16.0]
Cold weather	27/146 [18.5]	15/90 [16.7]	5/50 [10.0]	38/202 [18.8]	3/108 [2.8]	13/123 [10.6]	9/106 [8.5]	8/96 [8.3]	9/143 [6.3]	6/75 [8.0]
Humid weather	12/146 [8.2]	10/90 [11.1]	1/50 [2.0]	17/202 [8.4]	5/108 [4.6]	9/123 [7.3]	7/106 [6.6]	7/96 [7.3]	10/143 [7.0]	10/75 [13.3]
No movement	41/146 [28.1]	17/90 [18.9]	6/50 [12.0]	73/202 [36.1]	17/108 [15.7]	31/123 [25.2]	22/106 [20.8]	30/96 [31.3]	33/143 [23.1]	12/75 [16.0]
Movement	47/146 [32.2]	12/90 [13.3]	1/50 [2.0]	74/202 [36.6]	7/108 [6.5]	27/123 [22.0]	19/106 [17.9]	7/96 [7.3]	8/143 [5.6]	10/75 [13.3]
Sleep or rest	45/146 [30.8]	26/90 [28.9]	7/50 [14.0]	47/202 [23.3]	6/108 [5.6]	20/123 [16.3]	18/106 [17.0]	23/96 [24.0]	30/143 [21.0]	5/75 [6.7]
Urination or defecation	9/146 [6.2]	4/90 [4.4]	1/50 [2.0]	2/202 [1.0]	40/108 [37.0]	3/123 [2.4]	2/106 [1.9]	5/96 [5.2]	3/143 [2.1]	1/75 [1.3]
Negative mood/stress	26/146 [17.8]	19/90 [21.1]	16/50 [32.0]	41/202 [20.3]	14/108 [13.0]	19/123 [15.4]	14/106 [13.2]	14/96 [14.6]	13/143 [9.1]	12/75 [16.0]
Bright lights	6/146 [4.1]	5/90 [5.6]	3/50 [6.0]	5/202 [2.5]	1/108 [0.9]	5/123 [4.1]	2/106 [1.9]	2/96 [2.1]	1/143 [0.7]	2/75 [2.7]
Loud noises	5/146 [3.4]	3/90 [3.3]	6/50 [12.0]	6/202 [3.0]	2/108 [1.9]	3/123 [2.4]	3/106 [2.8]	3/96 [3.1]	1/143 [0.7]	1/75 [1.3]
Exercise	37/146 [25.3]	12/90 [13.3]	4/50 [8.0]	71/202 [35.1]	10/108 [9.3]	23/123 [18.7]	24/106 [22.6]	8/96 [8.3]	4/143 [2.8]	11/75 [14.7]
Fatigue	75/146 [51.4]	47/90 [52.2]	19/50 [38.0]	116/202 [57.4]	23/108 [21.3]	49/123 [39.8]	38/106 [35.8]	36/96 [37.5]	36/143 [25.2]	26/75 [34.7]
Other	9/146 [6.2]	9/90 [10.0]	4/50 [8.0]	14/202 [6.9]	12/108 [11.1]	7/123 [5.7]	9/106 [8.5]	3/96 [3.1]	6/143 [4.2]	2/75 [2.7]
*Ameliorating factors* ^a^										
Nothing/not sure	34/145 [23.4]	35/90 [38.9]	24/50 [48.0]	41/203 [20.2]	36/108 [33.3]	43/123 [35.0]	40/106 [37.7]	28/95 [29.5]	76/145 [52.4]	33/75 [44.0]
Eating	5/145 [3.4]	5/90 [5.6]	5/50 [10.0]	4/203 [2.0]	6/108 [5.6]	2/123 [1.6]	2/106 [1.9]	2/95 [2.1]	0/145 [0.0]	1/75 [1.3]
Speaking	1/145 [0.7]	3/90 [3.3]	5/50 [10.0]	2/203 [1.0]	0/108 [0.0]	0/123 [0.0]	0/106 [0.0]	0/95 [0.0]	0/145 [0.0]	0/75 [0.0]
Hot weather	7/145 [4.8]	4/90 [4.4]	2/50 [4.0]	14/203 [6.9]	2/108 [1.9]	8/123 [6.5]	3/106 [2.8]	3/95 [3.2]	2/145 [1.4]	1/75 [1.3]
Cold weather	3/145 [2.1]	3/90 [3.3]	1/50 [2.0]	4/203 [2.0]	0/108 [0.0]	1/123 [0.8]	5/106 [4.7]	3/95 [3.2]	5/145 [3.4]	4/75 [5.3]
Humid weather	0/145 [0.0]	2/90 [2.2]	1/50 [2.0]	5/203 [2.5]	1/108 [0.9]	3/123 [2.4]	1/106 [0.9]	0/95 [0.0]	1/145 [0.7]	1/75 [1.3]
No movement	16/145 [11.0]	3/90 [3.3]	3/50 [6.0]	36/203 [17.7]	7/108 [6.5]	16/123 [13.0]	7/106 [6.6]	4/95 [4.2]	8/145 [5.5]	4/75 [5.3]
Movement	49/145 [33.8]	26/90 [28.9]	10/50 [20.0]	65/203 [32.0]	22/108 [20.4]	35/123 [28.5]	30/106 [28.3]	30/95 [31.6]	39/145 [26.9]	14/75 [18.7]
Sleep or rest	50/145 [34.5]	26/90 [28.9]	10/50 [20.0]	90/203 [44.3]	24/108 [22.2]	49/123 [39.8]	31/106 [29.2]	28/95 [29.5]	24/145 [16.6]	22/75 [29.3]
Distraction	38/145 [26.2]	27/90 [30.0]	10/50 [20.0]	61/203 [30.0]	24/108 [22.2]	26/123 [21.1]	19/106 [17.9]	20/95 [21.1]	13/145 [9.0]	16/75 [21.3]
Urination or defecation	4/145 [2.8]	3/90 [3.3]	2/50 [4.0]	4/203 [2.0]	45/108 [41.7]	5/123 [4.1]	3/106 [2.8]	3/95 [3.2]	4/145 [2.8]	3/75 [4.0]
Exercise	45/145 [31.0]	29/90 [32.2]	9/50 [18.0]	70/203 [34.5]	23/108 [21.3]	30/123 [24.4]	26/106 [24.5]	20/95 [21.1]	24/145 [16.6]	14/75 [18.7]
Other	29/145 [20.0]	10/90 [11.1]	8/50 [16.0]	47/203 [23.2]	14/108 [13.0]	18/123 [14.6]	17/106 [16.0]	24/95 [25.3]	13/145 [9.0]	9/75 [12.0]

^a^ Multiple answers allowed.

For pain types where time dependent periods of pain accentuation were investigated in day and night times, the morning, evening, and night were frequently identified (over 15%) by participants in all pain types except RLS (only the evening [50/96, 52.1%] and night [70/96, 72.9%] were prominent). For nocturnal akinesia, where only nighttime periods were measured, the most frequently identified time dependent period of pain accentuation was the last third of the night (42/145, 29.0%), followed by the first (34/145, 23.4%) and middle (32/145, 22.1%) thirds. PD medication pharmacology also had an impact on pain. Wearing‐off or off periods were, in all pain types, the most frequently identified periods of PD medication dependent pain accentuation by participants (from [13/108, 12.0%] for abdominal visceral pain to [79/146, 54.1%] for dystonic pain). Peak‐ and early‐dose periods were also identified by participants (but at lower frequencies in all pain types as compared to wearing‐off or off periods) as periods of PD medication dependent pain accentuation (from [4/145, 2.8%] for nocturnal akinesia to [28/90, 31.1%] for dyskinetic pain). Many participants, however, reported that PD medication pharmacology made no difference to their pain (from [19/50, 38.0%] for temporomandibular pain to [81/108, 75.0%] for abdominal visceral pain).

No movement, movement, sleep or rest, negative mood/stress, and fatigue were frequently identified (over 15%) by participants as pain‐aggravating factors for most pain types, and so too, albeit only for a limited number of pain types, eating (abdominal visceral pain), hot weather (dystonic and central Parkinson's pain), cold weather (dystonic, dyskinetic, and musculoskeletal pain), urination or defecation (abdominal visceral pain), and exercise (dystonic, musculoskeletal, radicular neuropathic, and peripheral neuropathic pain). The most frequently identified (over 15%) pain‐relieving factors across all pain types by participants were movement, sleep or rest, distraction, and exercise. (One exception to this was nocturnal akinesia where distraction was only identified by [13/145, 9.0%] of participants.) Urination or defecation was also reported by a large proportion of participants (45/108, 41.7%) as a relieving factor for abdominal visceral pain.

#### 3.2.2 Pain Topography

Pain topography was only investigated for seven of the 10 pain types; temporomandibular and abdominal visceral pain, and nocturnal akinesia were omitted due to the self‐explanatory topography of these pain types (Table 3). The most frequently localized body segments by participants for dystonic and dyskinetic pain were the lower ([dystonic: foot/feet = 130/146, 70.5% and leg(s) = 100/146, 68.5%]; [dyskinetic: leg(s) 58/90, 64.4%]) and upper limbs ([dystonic: arm(s) = 42/146, 28.8%]; [dyskinetic: arm(s) = 36/90, 40.0%]) followed by the trunk (dystonic [30/146, 20.5%]; dyskinetic [25/90, 27.8%]). A large proportion of participants localized musculoskeletal pain in the back (148/203, 72.9%); however, all segments of the lower and upper limbs were also frequently identified (Table 3). The lower limbs were the most common site of pain identified by participants for most neurologically derived pain types: ([radicular neuropathic: leg(s) = 106/123, 86.2%]; [peripheral neuropathic: foot/feet = 84/106, 79.2% and leg(s) = 40/106, 37.7%]; [RLS: leg(s) = 95/96, 99.0%]). The lower limbs were also the most prominent site identified for central Parkinson's pain; however, it was also common in many other body segments (see Table 3). Excluding the response option ‘other’, pain was localized by participants in a median of 1 (dyskinetic pain and RLS) to 5 (musculoskeletal pain) body segments.

**TABLE 3:** Pain topography.

Dystonic^a^	Values (*n* [%])	Dyskinetic^a^	Values (*n* [%])	Musculoskeletal^a^	Values (*n* [%])	Radicular Neuropathic^a^	Values (*n* [%])	Peripheral Neuropathic^a^	Values (*n* [%])	Restless Legs syndrome^a^	Values (*n* [%])	Central Parkinson's^a^	Values (*n* [%])
Jaw/mouth/lower face	12/146 [8.2]	Head	22/90 [24.4]	Neck	105/203 [51.7]	Arm(s)	53/123 [43.1]	Foot/feet	84/106 [79.2]	Leg(s)	95/96 [99.0]	Face	10/75 [13.3]
Eye(s)	6/146 [4.1]	Arm(s)	36/90 [40.0]	Back	148/203 [72.9]	Leg(s)	106/123 [86.2]	Leg(s)	40/106 [37.7]	Arms(s)	11/96 [11.5]	Head	19/75 [25.3]
Throat/vocal cords	15/146 [10.3]	Trunk	25/90 [27.8]	Shoulder(s)	114/203 [56.2]	Chest	12/123 [9.8]	Hand(s)	38/106 [35.8]			Throat	7/75 [9.3]
Neck	35/146 [24.0]	Leg(s)	58/90 [64.4]	Upper arm(s)	56/203 [27.6]	Other	28/123 [22.8]	Arms(s)	17/106 [16.0]			Arm(s)	29/75 [38.7]
Arm(s)	42/146 [28.8]	Whole body	20/90 [22.2]	Elbow(s)	24/203 [11.8]			Other	10/106 [9.4]			Upper abdomen	25/75 [33.3]
Hand(s)	47/146 [32.2]	Other	9/90 [10.0]	Forearm(s)	44/203 [21.7]							Lower abdomen	27/75 [36.0]
Trunk	30/146 [20.5]			Wrist(s)	49/203 [24.1]							Rectum	5/75 [6.7]
Anus	3/146 [2.1]			Hand(s)	61/203 [30.0]							Genitals	14/75 [18.7]
Leg(s)	100/146 [68.5]			Hip(s)/pelvis	100/203 [49.3]							Leg(s)	38/75 [50.7]
Foot/feet	103/146 [70.5]			Upper leg(s)	60/203 [29.6]							Whole body	14/75 [18.7]
Other	13/146 [8.9]			Knee(s)	74/203 [36.5]							Other	8/75 [10.7]
				Calf/calves	69/203 [34.0]								
				Ankle(s)	60/203 [29.6]								
				Foot/feet	89/203 [43.8]								
				Other	10/203 [4.9]								
Total number of segments identified (median [IQR])^b^	3 [2–4]		1 [1–2]		5 [3–7]		2 [2–1]		2 [2–1]		1 [1–1]		4 [2–1]

^a^ Multiple answers allowed.

^b^ Does not include the response option 'other'.

#### 3.2.3 Pain Intensity

Pain intensity was measured for each pain type at its worst, at its least, and on average on a 10‐point scale, where 0 corresponded to no pain and 10 corresponded to pain as bad as you can imagine. Pain at its worst ranged from a median of 5 to 7, at its least from 1 to 2, and on average from 3 to 4 (Table 4).

**TABLE 4:** Pain intensity (0 = no pain, 10 = pain as bad as you can imagine).

	Pain intensity in the past month (median [IQR])		
	Pain at its worst	Pain at its least	Pain on average
Dystonic, *n* = 145	7 [5–8]	2 [1–3]	4 [2.5–5]
Dyskinetic, *n* = 90	6 [4–8]	2 [1–3]	4 [2–5]
Temporomandibular, *n* = 50	5 [3–7]	1 [1–2]	3 [1–5]
Musculoskeletal, *n* = 203	7 [6–8]	2 [1–3]	4 [3–6]
Abdominal visceral, *n* = 108	6 [4–8]	1 [1–2]	3 [2–5]
Radicular Neuropathic, *n* = 122	7 [5–8]	2 [1–3]	4 [2–5]
Peripheral Neuropathic, *n* = 105	6 [4–7]	2 [1–3]	3 [2–5]
Restless Legs Syndrome, *n* = 96	6 [3.25–7.75]	1 [1–2]	3 [2–5]
Nocturnal Akinesia, *n* = 143	5 [3–7]	1 [1–3]	3 [2–5]
Central Parkinson's, *n* = 74	6 [4–7]	1.5 [1–3]	3 [2–5]

## 4 Discussion

The aim of this study was to describe the characteristics of common pain types in people with PD. We found that pain was diverse in its presentation, with many participants experiencing multiple pain types in several different body segments at the same time. Adding to this complexity, acute attacks of pain were characteristic for many participants in most pain types and were typically frequent and prolonged. Periods of pain accentuation were most likely to occur in the morning, evening, or night and appeared to be associated with PD medication pharmacology and a variety of exacerbating and ameliorating factors. Fluctuations in pain were found to range from mild to severe in all pain types.

Consistent with previous reports from other regions, we found that musculoskeletal and dystonic pain were the most frequently declared pain types, and central and temporomandibular pain the least, with participants typically experiencing five pain types at the same time [27, 48, 49]. Direct comparisons of pain type frequencies, however, are complicated by differences in participant selection criteria and pain classifications systems employed in the literature. The King's Parkinson's Disease Pain Scale (KPPQ) and King's Parkinson's Disease Pain Questionnaire (KPPQ) (both developed and validated for PD) use a classification system of 14 pain types that is most comparable to this study [40, 50]. Research that has applied these instruments to PD patients with pain have reported similar, albeit moderately lower frequencies of pain types in participants than found in the present study [20, 48, 51, 52]. These differences may be principally explained by the broader and more detailed descriptions of pain types provided in the survey instrument used in this study as compared to the KPPS and KPPQ that are likely to result in a greater proportion of participants identifying their pain symptoms with a particular pain type.

Pain in most pain types was localized most frequently by participants in the lower limbs but was also reported to occur in almost any body part, from the head down to the feet, and even unusual body parts, including the eyes, throat, genitals, and rectum. Despite the paucity of research investigating the topography of dyskinetic, peripheral and radicular neuropathic, and central Parkinson's pain in PD, and differences in the division of body segments and pain classification systems used in previous studies, it may be concluded that the topography of pain found in this study is broadly in line with previous results [15, 21, 39, 53, 54, 55]. We also found that participants typically experienced pain in multiple body segments for a given pain type at the same time, and thus, the topographical data alone from this study highlights the diversity in the presentation of PD pain.

The pain categorization system used in this study is, to the authors' knowledge, the first PD specific instrument to include peripheral neuropathic pain as a specific pain type. Peripheral neuropathy in PD has only relatively recently been identified in the literature, with studies reporting an increased prevalence (30%–55%) as compared to controls (8%–14%) [56, 57, 58]. The development of peripheral neuropathy has been linked to prolonged levodopa exposure, abnormalities in vitamin B12, methylmalonic acid, and fasting homocysteine levels [31, 59]. The present study found that symptomatic peripheral neuropathy was declared by 41.9% of participants, strengthening the limited, but growing body of research in this area.

Acute attacks of pain were found in this study to be characteristic for many participants in most pain types (the temporal pattern of pain was not measured for nocturnal akinesia). These acute pain attacks were typically frequent (two to six times per week or more) and prolonged (minutes to hours) and were found to most likely to occur in the morning, evening, and/or night. Previous cross‐sectional studies have also reported high rates of fluctuating pain in PD patients but either did not differentiate between different pain types or used pain classification systems based on non‐specific terms (i.e., pain unrelated, related, or indirectly PD etc.) [17, 60]. Furthermore, fluctuating pain has previously been predominantly identified with only some PD pain types (i.e., dystonic, dyskinetic and central Parkinson's pain) [13, 14]. The pervasiveness of acute pain attacks found in this study in all pain types, is therefore, an important finding, particularly in view of previous research that has reported reduced quality of life in PD patients with fluctuating pain compared to those with non‐fluctuating pain [61].

Fluctuations in pain were also quantified numerically in this study on a 10‐point scale adapted from the Brief Pain Inventory [44]. Pain was found to be highest for dystonic, dyskinetic, musculoskeletal, and radicular neuropathic pain (4 on average) and lowest (3 on average) for the remaining pain types. In one of the few studies to investigate pain intensity in several PD pain types, Ozturk et al. [62], in a cross‐sectional study of PD patients (*N* = 73), found on a 0‐100 mm VAS scale, that pain intensity was highest in central Parkinson's pain (76/100), followed by dystonic (61/100), musculoskeletal (55/100) and radicular neuropathic (52/100). Sample sizes, however, were very small in some pain types (3 participants reported central Parkinson's pain, 11 dystonic, and 23 radicular) and the authors did not investigate fluctuations in pain intensity. Of note, in our study, dyskinetic pain intensity was rated in line with the other pain types by participants. The burden of dyskinesia in PD has recently been the topic of debate [35, 63] and there have been mixed reports of ‘painful dyskinesia’ in the literature. Most previous studies have either used the Unified Parkinson's Disease Scale (UPDRS) [64] or the KPPS to quantify dyskinetic pain. Studies that have used the UPDRS have found less than 6% of people with PD reported that their dyskinesias were painful [65, 66, 67]. While studies that have used the KPPS have reported the 9th highest intensity sub‐scores (severity [0–3] multiplied by frequency [0–4]) for dyskinetic pain out of the 14 PD pain types investigated by the KPPS, which was on par with visceral pain, a more well‐recognized PD pain type [10, 40, 51, 68]. Our research provides further evidence to suggest that dyskinesia is indeed a troublesome symptom for many people with PD.

A novel aspect of this research was that it investigated the relationship between PD medication pharmacology and pain in several specific PD pain types. While wearing‐off or off periods, and to a lesser extent, peak‐ and early‐dose periods were identified by participants as periods of pronounced pain, except for dystonic, dyskinetic, and temporomandibular pain, most reported that periods of pain accentuation were not influenced by PD pharmacology. Several plausible explanations may account for the different responses to PD medication found in this study; these include: dystonia, along with dyskinesia, is one of the main complications of levodopa therapy [69, 70]; the response to levodopa varies in people with PD [71]; there are several possible mechanisms underlying PD pain other than alterations in dopaminergic pathways [7]; and potential confounding factors, including concurrent analgesic use and comorbidities such as osteoarthritis. We do not, however, have an explanation for the degree of influence of dopaminergic medication on temporomandibular pain. There is presently a paucity of temporomandibular pain research in PD and further research is needed.

In addition, we found several pain exacerbating factors that may also contribute to temporal pain patterns, chief among these were no movement, movement, sleep or rest, negative mood/stress, and fatigue. Many of these factors have been associated with PD pain in previous studies [9, 72, 73, 74], however, one of the more notable aspects of the findings is the variability that was found both within and across pain types. No movement, for example, was frequently identified by patients as an exacerbating factor for many pain types, but so too was its opposing state, movement. Exercise, movement, and sleep or rest featured as both exacerbating and ameliorating factors in many pain types. Finally, only one exacerbating factor (fatigue) featured prominently in all pain types while others (i.e., urination or defecation, cold weather, and eating) were only prominent for one pain type. These finding suggest that here are different pathophysiological mechanisms, both within and across pain types, requiring different therapeutic approaches.

This study does have some limitations. The research relied on self‐reported online survey data, which is subject to inherent biases. In particular, temporal recall may be affected by recall bias, potentially influencing the findings related to the temporal patterns of pain. Furthermore, although participants were permitted to complete the survey with physical assistance and/or carer‐assisted if preferred, the online format may have excluded individuals with cognitive impairment, limited digital literacy, or those with more advanced PD. While the face and content validity of the survey instrument was established through pilot testing and expert review, neither it nor the novel pain classification system it used have undergone formal psychometric evaluation. The online design prevented verification of participants' self‐reported pain types, and despite efforts to distinguish between pain types, some overlap likely remained, particularly between dystonic and musculoskeletal pain, and peripheral and radicular neuropathic pain. Although survey instructions and branching logic were designed to guide participants toward selecting the most representative pain type, the potential for misclassification cannot be fully excluded. These limitations should be considered when interpreting the findings and future research should seek to validate the classification system and examine concordance between self‐reported and clinically determined pain types to enhance its reliability and applicability. Finally, recruitment relied on self‐selection and advocacy networks, potentially skewing the sample toward individuals who are more engaged or experiencing more severe pain.

## 5 Conclusion

This study makes a valuable contribution to the existing literature by providing the most detailed assessment of the topographical, sensory, and temporal characteristics of common PD pain types to date. By doing so it addresses critical knowledge gaps in the literature and its findings may be used to guide clinical diagnosis and treatment. Future studies may focus on longitudinal research to investigate how pain types and characteristics evolve over time in people with PD.

## Author Contributions

**Anthony Mezzini:** conceptualization, methodology, formal analysis, investigation, data curation, writing – original draft, writing – review and editing, visualization, project administration. **Saravana Kumar:** conceptualization, methodology, writing – review and editing, supervision. **Sue Sharrad:** conceptualization, methodology, writing – review and editing, supervision, funding acquisition. **Joanne Harmon:** conceptualization, writing – review and editing, supervision. **Marion Eckert:** conceptualization, writing – review and editing, supervision.

## Funding

This work was supported by Australian Government Research Training Program Scholarship and The Hospital Research Foundation Group—Parkinson’s.

## Ethics Statement

The study protocol was approved by the University of South Australia’s Human Research Ethics Committee on 9th November 2023 (ID: 205671).

## Consent

All participants provided informed written consent prior to participating in the study. No individuals under 18 years of age were permitted to participate.

## Conflicts of Interest

The authors declare no conflicts of interest.

## Supporting information


**Data S1:** papr70142‐sup‐0001‐Supinfo.docx.

## Data Availability

The data that support the findings of this study are available from the corresponding author upon reasonable request.
