# Supplementary material for: The Topographical, Sensory, and Temporal Characteristics of Pain in Parkinson’s Disease: A Cross‐Sectional Survey
Source: Pain Pract. 2026 Mar 11;26(4):e70142. doi: 10.1111/papr.70142 (PMC12979705; doi:10.1111/papr.70142)
Supplement: Supplementary file 1 — Data S1: papr70142‐sup‐0001‐Supinfo.docx. [file PAPR-26-0-s001.docx]

Supplementary Material 1

The Australian Parkinson’s Pain Study online survey instrument.

Start of Block: WELCOME STATEMENT

Q1.1 **WELCOME STATEMENT**

 Welcome to the **Australian Parkinson's Pain Study (APPS)** online survey of people with Parkinson's. The research team would like to thank you in advance for your time and effort participating in this study.

 The survey takes about 30 minutes to complete. To make it easier for you to participate, you can start the survey, stop and return to it later by clicking the survey link found on the APPS website (i.e., the same link you just used). This link will return you to the last question you answered in the survey. The answers to the questions you already provided will be automatically recorded/saved.

 Please note that if you would like to complete the survey over multiple sessions, **you will need to use the same device (i.e., the same computer or phone) and the same browser (i.e., Explorer, Safari, Chrome).**

 A caregiver or support person may help you complete the survey if you prefer.
 
You can go back and change your answers by using the **back button on the screen** but please do not use the "back" button on your internet browser.
 
Should you have any technical difficulties in completing the survey, please contact:
 
Anthony Mezzini
Principal Investigator
Ph: 0427 820 256
Email: anthony.mezzini@mymail.unisa.edu.au

End of Block: WELCOME STATEMENT

Start of Block: INFORMATION FOR PARTICIPANTS

Q2.1 **INFORMATION FOR PARTICIPANTS**

 Please read the attached participant information sheet.

 Participant Information Sheet

Q2.2 **PARTICIPANT CONSENT**

 By completing and submitting the survey, you are indicating that you have read and understood the participant information sheet and give your consent to be involved in the study.

- Agree and continue (1)

End of Block: INFORMATION FOR PARTICIPANTS

Start of Block: ELIGIBILITY CRITERIA CHECKLIST

Q3.1 **ELIGIBILITY CRITERIA CHECKLIST**

 We would like to ask a few questions to determine if you meet the eligibility criteria for this study.

Q3.2 Have you been diagnosed with Parkinson’s by a neurologist or movement disorder specialist?

- Yes (1)
- No (2)

Q3.3 Have you experienced **any** pain symptoms in the past month (e.g. joint and muscle pain, central Parkinson's pain, muscle cramps/dystonia, abdominal pain)?

- Yes (1)
- No (2)

Q3.4 Are you an Australian citizen or permanent resident currently living in Australia?

- Yes (1)
- No (2)

Q3.5 Are you 18 years of age or older?

- Yes (1)
- No (2)

End of Block: ELIGIBILITY CRITERIA CHECKLIST

Start of Block: DEMOGRAPHIC INFORMATION

Q4.1 **DEMOGRAPHIC & HEALTH INFORMATION**

 We would like to collect some basic demographic and health information. Your responses to these questions will help us to interpret responses to the other questions.

Q4.2 What is your sex?

- Male (1)
- Female (2)
- Other (3)
- Prefer not to say (4)

| 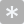 |
| --- |

Q4.3 What is your current age (in years)?

________________________________________________________________

| 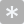 |
| --- |

Q4.4 What is the postcode where you live most of the time?

________________________________________________________________

| 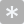 |
| --- |

Q4.5 What year where you diagnosed with Parkinson’s? If you can’t remember, please make your best estimate.

________________________________________________________________

Q4.6 Which statement best describes your current symptoms?

- Stage 1: I have mild symptoms that do not generally interfere with daily activities. Movement symptoms only affect one side of my body. (1)
- Stage 2: Movement symptoms mildly affect both sides of my body. I have no problems with my balance, but daily tasks are more difficult and take longer. (2)
- Stage 3: Movement symptoms moderately affect both sides of my body and I have problems with my balance. I am somewhat restricted in my daily activities, but I am capable of leading an independent life. (3)
- Stage 4: I am severely limited. However, I am still able to stand and walk without assistance, but I may need a cane/walker for safety. I need help with daily activities and I am unable to live alone. (4)
- Stage 5: I am bound to a wheelchair or bedridden, unless I receive assistance. (5)

Q4.7 Which of the following medical doctors/specialists is most responsible for your Parkinson’s treatment?

- Neurologist (1)
- Geriatrician (3)
- General Practitioner (GP) (4)
- Other (please specify) (5) __________________________________________________

End of Block: DEMOGRAPHIC INFORMATION

Start of Block: INTRO TO CHARACTERISTICS OF PAIN SYMPTOMS

Q5.1 **SECTION A : CHARACTERISTICS OF PAIN SYMPTOMS**

 The following section asks about 10 different types of pain symptoms that are commonly experienced by people with Parkinson’s (e.g. joint and muscle pain, central Parkinson's pain, muscle cramps/dystonia, abdominal pain).

 We refer to these pain symptoms as **'Parkinson's related pain'**.

End of Block: INTRO TO CHARACTERISTICS OF PAIN SYMPTOMS

Start of Block: CHARACTERISTICS OF PAIN SYMPTOMS - DYSTONIC PAIN

Q6.1 **SUB-SECTION A.1 : DYSTONIC PAIN**

 This sub-section asks about pain related to **involuntary muscle contractions/cramps** that are often very forceful and may cause an **abnormal posture and/or curling/twisting of body parts**.

 It can affect any body part, but commonly affects the arms, legs, hands, and feet.

 We refer to this type of pain as dystonic pain.

Q6.2 In the past month, have you experienced dystonic pain?

- Yes (1)
- No (2)

Skip To: End of Block If Q6.2 = No

| Page Break |  |
| --- | --- |

Q6.3 Please indicate the area(s) of the body where your dystonic pain occurs. (Select all that apply)

- Jaw/mouth/lower face (1)
- Eye(s) (2)
- Throat/vocal cords (12)
- Neck (3)
- Arm(s) (5)
- Hand(s) (6)
- Trunk (7)
- Anus (11)
- Leg(s) (8)
- Foot/feet (9)
- Other (please specify) (10) __________________________________________________

Q6.4 How long have you had dystonic pain?

- Less than 3 months (8)
- 3 months to less than 1 year (9)
- More than 1 year (10)

Q6.5 Which of the following descriptions best describes your dystonic pain in the past month?

- Continuous pain with slight variations (1)
- Continuous pain with large variations (2)
- Acute attacks of pain with pain free periods in-between (3)
- Acute attacks of pain on a background of constant pain (4)

Skip To: Q6.8 If Q6.5 = Continuous pain with slight variations

Skip To: Q6.8 If Q6.5 = Continuous pain with large variations

| Page Break |  |
| --- | --- |

Q6.6 On average, how often do you experience acute dystonic pain attacks?

- Several times a day (1)
- Once a day (8)
- Two to six times a week (9)
- Once a week (10)
- Less than once a week (11)

Q6.7 On average, how long do your acute dystonic pain attacks last?

- Seconds (1)
- Minutes (2)
- Hours (3)
- Days (4)

| Page Break |  |
| --- | --- |

Q6.8 Is your dystonic pain **WORSE**... (Select all that apply)

- In the morning (3)
- At noon (4)
- In the afternoon (5)
- In the evening (6)
- At night (7)
- No difference (8)

Q6.9 Is your dystonic pain **WORSE** during... (Select all that apply)

- **Peak-dose periods** when your Parkinson’s medication is most effective (30-90 mins after medication consumption) (1)
- **Wearing-off or off periods** when your Parkinson’s medication is not working (4)
- **Early-dose periods** (10-30 mins after medication consumption) (5)
- No difference (3)
- I don’t take Parkinson’s medication (6)

| Page Break |  |
| --- | --- |

Q6.10 What factors, if any, increase and/or trigger your dystonic pain? (Select all that apply)

- Nothing/not sure (1)
- Eating (6)
- Speaking (7)
- Hot weather (8)
- Cold weather (9)
- Humid weather (10)
- No movement (13)
- Movement (14)
- Sleep or rest (15)
- Urination or defecation (17)
- Negative mood/stress (18)
- Bright lights (20)
- Loud noises (21)
- Exercise (24)
- Fatigue (25)
- Other (please specify) (26) __________________________________________________

| Page Break |  |
| --- | --- |

Q6.11 What factors, if any, decrease your dystonic pain? (Select all that apply)

- Nothing/not sure (1)
- Eating (6)
- Speaking (7)
- Hot weather (8)
- Cold weather (9)
- Humid weather (10)
- No movement (12)
- Movement (13)
- Sleep or rest (14)
- Distraction (e.g. TV, reading, music) (16)
- Urination or defecation (17)
- Exercise (20)
- Other (please specify) (21) __________________________________________________

| Page Break |  |
| --- | --- |

Q6.12 Please rate your dystonic pain by selecting the one number that best describes your pain in the past month: 

 **(0 = no pain : 10 = pain as bad as you can imagine)**

|  | 0 | 1 | 2 | 3 | 4 | 5 | 6 | 7 | 8 | 9 | 10 |
| --- | --- | --- | --- | --- | --- | --- | --- | --- | --- | --- | --- |

| PAIN AT ITS **WORST** (1) |  |
| --- | --- |
| PAIN AT ITS **LEAST** (2) |  |
| PAIN ON **AVERAGE** (3) |  |

End of Block: CHARACTERISTICS OF PAIN SYMPTOMS - DYSTONIC PAIN

Start of Block: CHARACTERISTICS OF PAIN SYMPTOMS - DYSKINETIC PAIN

Q7.1 **SUB-SECTION A.2 : DYSKINETIC PAIN**

 This sub-section asks about pain related to **abnormal, involuntary, erratic, wriggling/writhing or twitch-like flowing motion** of the arms, legs, head, trunk, or whole body that is associated with levodopa use.

 We refer to this type of pain as dyskinetic pain.

 NOTE: Dyskinesia should not be confused with tremor.

Q7.2 In the past month, have you experienced dyskinetic pain?

- Yes (1)
- No (2)

Skip To: End of Block If Q7.2 = No

| Page Break |  |
| --- | --- |

Q7.3 Please indicate the area(s) of the body where your dyskinetic pain occurs. (Select all that apply)

- Head (1)
- Arm(s) (2)
- Trunk (12)
- Leg(s) (5)
- Whole body (6)
- Other (please specify) (10) __________________________________________________

Q7.4 How long have you had dyskinetic pain?

- Less than 3 months (8)
- 3 months to less than 1 year (9)
- More than 1 year (10)

Q7.5 Which of the following descriptions best describes your dyskinetic pain in the past month?

- Continuous pain with slight variations (1)
- Continuous pain with large variations (2)
- Acute attacks of pain with pain free periods in-between (3)
- Acute attacks of pain on a background of constant pain (4)

Skip To: Q7.8 If Q7.5 = Continuous pain with slight variations

Skip To: Q7.8 If Q7.5 = Continuous pain with large variations

| Page Break |  |
| --- | --- |

Q7.6 On average, how often do you experience acute dyskinetic pain attacks?

- Several times a day (1)
- Once a day (8)
- Two to six times a week (9)
- Once a week (10)
- Less than once a week (11)

Q7.7 On average, how long do your acute dyskinetic pain attacks last?

- Seconds (1)
- Minutes (2)
- Hours (3)
- Days (4)

| Page Break |  |
| --- | --- |

Q7.8 Is your dyskinetic pain **WORSE**... (Select all that apply)

- In the morning (3)
- At noon (4)
- In the afternoon (5)
- In the evening (6)
- At night (7)
- No difference (8)

Q7.9 Is your dyskinetic pain **WORSE** during... (Select all that apply)

- **Peak-dose** **periods** when your Parkinson’s medication is most effective (30-90 mins after medication consumption) (1)
- **Wearing-off or off periods** when your Parkinson’s medication is not working (4)
- **Early-dose periods** (10-30 mins after medication consumption) (5)
- No difference (3)
- I don’t take Parkinson’s medication (6)

| Page Break |  |
| --- | --- |

Q7.10 What factors, if any, increase and/or trigger your dyskinetic pain? (Select all that apply)

- Nothing/not sure (1)
- Eating (6)
- Speaking (7)
- Hot weather (8)
- Cold weather (9)
- Humid weather (10)
- No movement (13)
- Movement (14)
- Sleep or rest (15)
- Urination or defecation (17)
- Negative mood/stress (18)
- Bright lights (20)
- Loud noises (21)
- Exercise (24)
- Fatigue (25)
- Other (please specify) (26) __________________________________________________

| Page Break |  |
| --- | --- |

Q7.11 What factors, if any, decrease your dyskinetic pain? (Select all that apply)

- Nothing/not sure (1)
- Eating (6)
- Speaking (7)
- Hot weather (8)
- Cold weather (9)
- Humid weather (10)
- No movement (12)
- Movement (13)
- Sleep or rest (14)
- Distraction (e.g. TV, reading, music) (16)
- Urination or defecation (17)
- Exercise (20)
- Other (please specify) (21) __________________________________________________

| Page Break |  |
| --- | --- |

Q7.12 Please rate your dyskinetic pain by selecting the one number that best describes your pain in the past month: 

 **(0 = no pain : 10 = pain as bad as you can imagine)**

|  | 0 | 1 | 2 | 3 | 4 | 5 | 6 | 7 | 8 | 9 | 10 |
| --- | --- | --- | --- | --- | --- | --- | --- | --- | --- | --- | --- |

| PAIN AT ITS **WORST** (1) |  |
| --- | --- |
| PAIN AT ITS **LEAST** (2) |  |
| PAIN ON **AVERAGE** (3) |  |

End of Block: CHARACTERISTICS OF PAIN SYMPTOMS - DYSKINETIC PAIN

Start of Block: CHARACTERISTICS OF PAIN SYMPTOMS - TEMPOROMANDIBULAR PAIN

Q8.1 **SUB-SECTION A.3 : TEMPOROMANDIBULAR PAIN**

 This sub-section asks about pain that occurs around the **muscles and/or joint of the jaw** that is related to grinding/clenching your teeth and/or chewing.

 We refer to this type of pain as temporomandibular pain.

 NOTE: The jaw joint is located in front of the ear.

Q8.2 In the past month, have you experienced temporomandibular pain?

- Yes (1)
- No (2)

Skip To: End of Block If Q8.2 = No

| Page Break |  |
| --- | --- |

Q8.3 How long have you had temporomandibular pain?

- Less than 3 months (8)
- 3 months to less than 1 year (9)
- More than 1 year (10)

Q8.4 Which of the following descriptions best describes your temporomandibular pain in the past month?

- Continuous pain with slight variations (8)
- Continuous pain with large variations (9)
- Acute attacks of pain with pain free periods in-between (11)
- Acute attacks of pain on a background of constant pain (10)

Skip To: Q8.7 If Q8.4 = Continuous pain with slight variations

Skip To: Q8.7 If Q8.4 = Continuous pain with large variations

| Page Break |  |
| --- | --- |

Q8.5 On average, how often do you experience acute temporomandibular pain attacks?

- Several times a day (1)
- Once a day (8)
- Two to six times a week (9)
- Once a week (10)
- Less than once a week (11)

Q8.6 On average, how long do your acute temporomandibular pain attacks last?

- Seconds (1)
- Minutes (8)
- Hours (9)
- Days (10)

| Page Break |  |
| --- | --- |

Q8.7 Is your temporomandibular pain **WORSE**... (Select all that apply)

- In the morning (3)
- At noon (4)
- In the afternoon (5)
- In the evening (6)
- At night (7)
- No difference (8)

Q8.8 Is your temporomandibular pain **WORSE** during... (Select all that apply)

- **Peak-dose** **periods** when your Parkinson’s medication is most effective (30-90 mins after medication consumption) (1)
- **Wearing-off** **or off periods** when your Parkinson’s medication is not working (4)
- **Early-dose** **periods** (10-30 mins after medication consumption) (5)
- No difference (6)
- I don't take Parkinson's medication (7)

| Page Break |  |
| --- | --- |

Q8.9 What factors, if any, increase and/or trigger your temporomandibular pain? (Select all that apply)

- Nothing/not sure (1)
- Eating (6)
- Speaking (7)
- Hot weather (8)
- Cold weather (9)
- Humid weather (10)
- No movement (13)
- Movement (14)
- Sleep or rest (15)
- Urination or defecation (17)
- Negative mood/stress (18)
- Bright lights (20)
- Loud noises (21)
- Exercise (24)
- Fatigue (25)
- Other (please specify) (26) __________________________________________________

| Page Break |  |
| --- | --- |

Q8.10 What factors, if any, decrease your temporomandibular pain? (Select all that apply)

- Nothing/not sure (1)
- Eating (6)
- Speaking (7)
- Hot weather (8)
- Cold weather (9)
- Humid weather (10)
- No movement (12)
- Movement (13)
- Sleep or rest (14)
- Distraction (e.g. TV, reading, music) (16)
- Urination or defecation (17)
- Exercise (20)
- Other (please specify) (21) __________________________________________________

| Page Break |  |
| --- | --- |

Q8.11 Please rate your temporomandibular pain by selecting the one number that best describes your pain in the past month: 

 **(0 = no pain : 10 = pain as bad as you can imagine)**

|  | 0 | 1 | 2 | 3 | 4 | 5 | 6 | 7 | 8 | 9 | 10 |
| --- | --- | --- | --- | --- | --- | --- | --- | --- | --- | --- | --- |

| PAIN AT ITS **WORST** (1) |  |
| --- | --- |
| PAIN AT ITS **LEAST** (2) |  |
| PAIN ON **AVERAGE** (3) |  |

End of Block: CHARACTERISTICS OF PAIN SYMPTOMS - TEMPOROMANDIBULAR PAIN

Start of Block: CHARACTERISTICS OF PAIN SYMPTOMS - MUSCULOSKELETAL PAIN

Q9.1 **SUB-SECTION A.4 : MUSCULOSKELETAL PAIN**

 This sub-section asks about pain that occurs **around the muscles and/or joints** of the **neck, back, shoulders, arms, hands, hips/pelvis, legs, and feet** in the absence of painful dystonic muscle cramps and dyskinesia.

 We refer to this type of pain as musculoskeletal pain and is often related to muscle stiffness/rigidity, slowness of movement and/or abnormal posture.

Q9.2 In the past month, have you experienced musculoskeletal pain?

- Yes (1)
- No (2)

Skip To: End of Block If Q9.2 = No

| Page Break |  |
| --- | --- |

Q9.3 Please indicate the area(s) of the body where your musculoskeletal pain occurs. (Select all that apply)

- Neck (13)
- Back (28)
- Shoulder(s) (29)
- Upper arm(s) (31)
- Elbow(s) (32)
- Forearm(s) (33)
- Wrist(s) (34)
- Hand(s) (35)
- Hip(s)/pelvis (37)
- Upper leg(s) (39)
- Knee(s) (40)
- Calf/calves (41)
- Ankle(s) (42)
- Foot/feet (45)
- Other (please specify) (10) __________________________________________________

| Page Break |  |
| --- | --- |

Q9.4 How long have you had musculoskeletal pain?

- Less than 3 months (2)
- 3 months to less than 1 year (3)
- More than 1 year (7)

Q9.5 Which of the following descriptions best describes your musculoskeletal pain in the past month?

- Continuous pain with slight variations (1)
- Continuous pain with large variations (2)
- Acute attacks of pain with pain free periods in-between (3)
- Acute attacks of pain on a background of constant pain (4)

Skip To: Q9.8 If Q9.5 = Continuous pain with slight variations

Skip To: Q9.8 If Q9.5 = Continuous pain with large variations

| Page Break |  |
| --- | --- |

Q9.6 On average, how often do you experience acute musculoskeletal pain attacks?

- Several times a day (1)
- Once a day (2)
- Two to six times a week (3)
- Once a week (4)
- Less than once a week (7)

Q9.7 On average, how long do your acute musculoskeletal pain attacks last?

- Seconds (1)
- Minutes (2)
- Hours (3)
- Days (4)

| Page Break |  |
| --- | --- |

Q9.8 Is your musculoskeletal pain **WORSE**... (Select all that apply)

- In the morning (3)
- At noon (4)
- In the afternoon (5)
- In the evening (6)
- At night (7)
- No difference (8)

Q9.9 Is your musculoskeletal pain **WORSE** during... (Select all that apply)

- **Peak-dose** **periods** when your Parkinson’s medication is most effective (30-90 mins after medication consumption) (1)
- **Wearing-off or off periods** when your Parkinson’s medication is not working (4)
- **Early-dose** **periods** (10-30 mins after medication consumption) (5)
- No difference (6)
- I don’t take Parkinson’s medication (7)

| Page Break |  |
| --- | --- |

Q9.10 What factors, if any, increase and/or trigger your musculoskeletal pain? (Select all that apply)

- Nothing/not sure (1)
- Eating (6)
- Speaking (7)
- Hot weather (8)
- Cold weather (9)
- Humid weather (10)
- No movement (13)
- Movement (14)
- Sleep or rest (15)
- Urination or defecation (17)
- Negative mood/stress (18)
- Bright lights (20)
- Loud noises (21)
- Exercise (24)
- Fatigue (25)
- Other (please specify) (26) __________________________________________________

| Page Break |  |
| --- | --- |

Q9.11 What factors, if any, decrease your musculoskeletal pain? (Select all that apply)

- Nothing/not sure (1)
- Eating (6)
- Speaking (7)
- Hot weather (8)
- Cold weather (9)
- Humid weather (10)
- No movement (12)
- Movement (13)
- Sleep or rest (14)
- Distraction (e.g. TV, reading, music) (16)
- Urination or defecation (17)
- Exercise (20)
- Other (please specify) (21) __________________________________________________

| Page Break |  |
| --- | --- |

Q9.12 Please rate your musculoskeletal pain by selecting the one number that best describes your pain in the past month: 

 **(0 = no pain : 10 = pain as bad as you can imagine)**

|  | 0 | 1 | 2 | 3 | 4 | 5 | 6 | 7 | 8 | 9 | 10 |
| --- | --- | --- | --- | --- | --- | --- | --- | --- | --- | --- | --- |

| PAIN AT ITS **WORST** (1) |  |
| --- | --- |
| PAIN AT ITS **LEAST** (2) |  |
| PAIN ON **AVERAGE** (3) |  |

End of Block: CHARACTERISTICS OF PAIN SYMPTOMS - MUSCULOSKELETAL PAIN

Start of Block: CHARACTERISTICS OF PAIN SYMPTOMS - ABDOMINAL VISCERAL PAIN

Q10.1 **SUB-SECTION A.5 : ABDOMINAL VISCERAL PAIN**

 This sub-section asks about pain that occurs in the **stomach and/or bowels** that is related to constipation and/or gastroparesis (nausea, sensation of fullness, stomach pain and/or bloating).

 We refer to this type of pain as abdominal visceral pain.

Q10.2 In the past month, have you experienced abdominal visceral pain?

- Yes (1)
- No (2)

Skip To: End of Block If Q10.2 = No

| Page Break |  |
| --- | --- |

Q10.3 How long have you had abdominal visceral pain?

- Less than 3 months (2)
- 3 months to less than 1 year (3)
- More than 1 year (4)

Q10.4 Which of the following descriptions best describes your abdominal visceral pain in the past month?

- Continuous pain with slight variations (1)
- Continuous pain with large variations (2)
- Acute attacks of pain with pain free periods in-between (3)
- Acute attacks of pain on a background of constant pain (4)

Skip To: Q10.7 If Q10.4 = Continuous pain with slight variations

Skip To: Q10.7 If Q10.4 = Continuous pain with large variations

| Page Break |  |
| --- | --- |

Q10.5 On average, how often do you experience acute abdominal visceral pain attacks?

- Several times a day (1)
- Once a day (2)
- Two to six times a week (3)
- Once a week (4)
- Less than once a week (8)

Q10.6 On average, how long do your acute abdominal visceral pain attacks last?

- Seconds (1)
- Minutes (2)
- Hours (3)
- Days (4)

| Page Break |  |
| --- | --- |

Q10.7 Is your abdominal visceral pain **WORSE**... (Select all that apply)

- In the morning (3)
- At noon (4)
- In the afternoon (5)
- In the evening (6)
- At night (7)
- No difference (8)

Q10.8 Is your abdominal visceral pain **WORSE** during... (Select all that apply)

- **Peak-dose** **periods** when your Parkinson’s medication is most effective (30-90 mins after medication consumption) (1)
- **Wearing-off** **or off periods** when your Parkinson’s medication is not working (4)
- **Early-dose** **periods** (10-30 mins after medication consumption) (5)
- No difference (6)
- I don’t take Parkinson’s medication (7)

| Page Break |  |
| --- | --- |

Q10.9 What factors, if any, increase and/or trigger your abdominal visceral pain? (Select all that apply)

- Nothing/not sure (1)
- Eating (6)
- Speaking (7)
- Hot weather (8)
- Cold weather (9)
- Humid weather (10)
- No movement (13)
- Movement (14)
- Sleep or rest (15)
- Urination or defecation (17)
- Negative mood/stress (18)
- Bright lights (20)
- Loud noises (21)
- Exercise (24)
- Fatigue (25)
- Other (please specify) (26) __________________________________________________

| Page Break |  |
| --- | --- |

Q10.10 What factors, if any, decrease your abdominal visceral pain? (Select all that apply)

- Nothing/not sure (1)
- Eating (6)
- Speaking (7)
- Hot weather (8)
- Cold weather (9)
- Humid weather (10)
- No movement (12)
- Movement (13)
- Sleep or rest (14)
- Distraction (e.g. TV, reading, music) (16)
- Urination or defecation (17)
- Exercise (20)
- Other (please specify) (21) __________________________________________________

| Page Break |  |
| --- | --- |

Q10.11 Please rate your abdominal visceral pain by selecting the one number that best describes your pain in the past month: 

 **(0 = no pain : 10 = pain as bad as you can imagine)**

|  | 0 | 1 | 2 | 3 | 4 | 5 | 6 | 7 | 8 | 9 | 10 |
| --- | --- | --- | --- | --- | --- | --- | --- | --- | --- | --- | --- |

| PAIN AT ITS **WORST** (1) |  |
| --- | --- |
| PAIN AT ITS **LEAST** (2) |  |
| PAIN ON **AVERAGE** (3) |  |

End of Block: CHARACTERISTICS OF PAIN SYMPTOMS - ABDOMINAL VISCERAL PAIN

Start of Block: CHARACTERISTICS OF PAIN SYMPTOMS - RADICULAR NEUROPATHIC PAIN

Q11.1 **SUB-SECTION A.6 : RADICULAR NEUROPATHIC PAIN**

 This sub-section asks about **shooting/radiating pain, numbness, weakness and/or tingling** down the **arms, legs, and/or into the chest** that may be accompanied with pain in your neck or back.

 We refer to this type of pain as radicular neuropathic pain.

 NOTE: When radicular neuropathic pain occurs in the legs, it is sometimes called sciatica.

Q11.2 In the past month, have you experienced radicular neuropathic pain?

- Yes (1)
- No (2)

Skip To: End of Block If Q11.2 = No

| Page Break |  |
| --- | --- |

Q11.3 Please indicate the area(s) of the body where your radicular neuropathic pain occurs? (Select all that apply)

- Arm(s) (1)
- Leg(s) (2)
- Chest (3)
- Other (please specify) (4) __________________________________________________

Q11.4 How long have you had radicular neuropathic pain?

- Less than 3 months (8)
- 3 months to less than 1 year (9)
- More than 1 year (10)

Q11.5 Which of the following descriptions best describes your radicular neuropathic pain in the past month?

- Continuous pain with slight variations (1)
- Continuous pain with large variations (2)
- Acute attacks of pain with pain free periods in-between (3)
- Acute attacks of pain on a background of constant pain (4)

Skip To: Q11.8 If Q11.5 = Continuous pain with slight variations

Skip To: Q11.8 If Q11.5 = Continuous pain with large variations

| Page Break |  |
| --- | --- |

Q11.6 On average, how often do you experience acute radicular neuropathic pain attacks?

- Several times a day (1)
- Once a day (8)
- Two to six times a week (9)
- Once a week (10)
- Less than once a week (11)

Q11.7 On average, how long do your acute radicular neuropathic pain attacks last?

- Seconds (1)
- Minutes (2)
- Hours (3)
- Days (4)

| Page Break |  |
| --- | --- |

Q11.8 Is your radicular neuropathic pain **WORSE**... (Select all that apply)

- In the morning (3)
- At noon (4)
- In the afternoon (5)
- In the evening (6)
- At night (7)
- No difference (8)

Q11.9 Is your radicular neuropathic pain **WORSE** during... (Select all that apply)

- **Peak-dose periods** when your Parkinson’s medication is most effective (30-90 mins after medication consumption) (1)
- **Wearing-off or off periods** when your Parkinson’s medication is not working (4)
- **Early-dose periods** (10-30 mins after medication consumption) (5)
- No difference (3)
- I don’t take Parkinson’s medication (6)

| Page Break |  |
| --- | --- |

Q11.10 What factors, if any, increase and/or trigger your radicular neuropathic pain? (Select all that apply)

- Nothing/not sure (1)
- Eating (6)
- Speaking (7)
- Hot weather (8)
- Cold weather (9)
- Humid weather (10)
- No movement (13)
- Movement (14)
- Sleep or rest (15)
- Urination or defecation (17)
- Negative mood/stress (18)
- Bright lights (20)
- Loud noises (21)
- Exercise (24)
- Fatigue (25)
- Other (please specify) (26) __________________________________________________

| Page Break |  |
| --- | --- |

Q11.11 What factors, if any, decrease your radicular neuropathic pain? (Select all that apply)

- Nothing/not sure (1)
- Eating (6)
- Speaking (7)
- Hot weather (8)
- Cold weather (9)
- Humid weather (10)
- No movement (12)
- Movement (13)
- Sleep or rest (14)
- Distraction (e.g. TV, reading, music) (16)
- Urination or defecation (17)
- Exercise (20)
- Other (please specify) (21) __________________________________________________

| Page Break |  |
| --- | --- |

Q11.12 Please rate your radicular neuropathic pain by selecting the one number that best describes your pain in the past month: 

 **(0 = no pain : 10 = pain as bad as you can imagine)**

|  | 0 | 1 | 2 | 3 | 4 | 5 | 6 | 7 | 8 | 9 | 10 |
| --- | --- | --- | --- | --- | --- | --- | --- | --- | --- | --- | --- |

| PAIN AT ITS **WORST** (1) |  |
| --- | --- |
| PAIN AT ITS **LEAST** (2) |  |
| PAIN ON **AVERAGE** (3) |  |

End of Block: CHARACTERISTICS OF PAIN SYMPTOMS - RADICULAR NEUROPATHIC PAIN

Start of Block: CHARACTERISTICS OF PAIN SYMPTOMS - PERIPHERAL NEUROPATHIC PAIN

Q12.1 **SUB-SECTION A.7 : PERIPHERAL NEUROPATHIC PAIN**

 This sub-section asks about **burning pain, tingling and/or numbness** in the **feet and/or hands** that may extend into the lower portion of the limbs.

 We refer to this type of pain as peripheral neuropathic pain.

 NOTE: Peripheral neuropathic pain should **not** be confused with radicular neuropathic pain that causes a shooting/radiating pain down the arm, legs, and/or into the chest.

Q12.2 In the past month, have you experienced peripheral neuropathic pain?

- Yes (1)
- No (2)

Skip To: End of Block If Q12.2 = No

| Page Break |  |
| --- | --- |

Q12.3 Please indicate the area(s) of the body where your peripheral neuropathic pain occurs. (Select all that apply)

- Foot/feet (1)
- Leg(s) (3)
- Hand(s) (4)
- Arm(s) (5)
- Other (please specify) (2) __________________________________________________

Q12.4 How long have you had peripheral neuropathic pain?

- Less than 3 months (8)
- 3 months to less than 1 year (9)
- More than 1 year (10)

Q12.5 Which of the following descriptions best describes your peripheral neuropathic pain in the past month?

- Continuous pain with slight variations (1)
- Continuous pain with large variations (2)
- Acute attacks of pain with pain free periods in-between (3)
- Acute attacks of pain on a background of constant pain (4)

Skip To: Q12.8 If Q12.5 = Continuous pain with slight variations

Skip To: Q12.8 If Q12.5 = Continuous pain with large variations

| Page Break |  |
| --- | --- |

Q12.6 On average, how often do you experience acute peripheral neuropathic pain attacks?

- Several times a day (1)
- Once a day (8)
- Two to six times a week (9)
- Once a week (10)
- Less than once a week (11)

Q12.7 On average, how long do your acute peripheral neuropathic pain attacks last?

- Seconds (1)
- Minutes (2)
- Hours (3)
- Days (4)

| Page Break |  |
| --- | --- |

Q12.8 Is your peripheral neuropathic pain **WORSE**... (Select all that apply)

- In the morning (3)
- At noon (4)
- In the afternoon (5)
- In the evening (6)
- At night (7)
- No difference (8)

Q12.9 Is your peripheral neuropathic pain **WORSE** during... (Select all that apply)

- **Peak-dose** **periods** when your Parkinson’s medication is most effective (30-90 mins after medication consumption) (1)
- **Wearing-off** **or off periods** when your Parkinson’s medication is not working (4)
- **Early-dose** **periods** (10-30 mins after medication consumption) (5)
- No difference (3)
- I don’t take Parkinson’s medication (6)

| Page Break |  |
| --- | --- |

Q12.10 What factors, if any, increase and/or trigger your peripherial neuropathic pain? (Select all that apply)

- Nothing/not sure (1)
- Eating (6)
- Speaking (7)
- Hot weather (8)
- Cold weather (9)
- Humid weather (10)
- No movement (13)
- Movement (14)
- Sleep or rest (15)
- Urination or defecation (17)
- Negative mood/stress (18)
- Bright lights (20)
- Loud noises (21)
- Exercise (24)
- Fatigue (25)
- Other (please specify) (26) __________________________________________________

| Page Break |  |
| --- | --- |

Q12.11 What factors, if any, decrease your peripheral neuropathic pain? (Select all that apply)

- Nothing/not sure (1)
- Eating (6)
- Speaking (7)
- Hot weather (8)
- Cold weather (9)
- Humid weather (10)
- No movement (12)
- Movement (13)
- Sleep or rest (14)
- Distraction (e.g. TV, reading, music) (16)
- Urination or defecation (17)
- Exercise (20)
- Other (please specify) (21) __________________________________________________

| Page Break |  |
| --- | --- |

Q12.12 Please rate your peripheral neuropathic pain by selecting the one number that best describes your pain in the past month: 

 **(0 = no pain : 10 = pain as bad as you can imagine)**

|  | 0 | 1 | 2 | 3 | 4 | 5 | 6 | 7 | 8 | 9 | 10 |
| --- | --- | --- | --- | --- | --- | --- | --- | --- | --- | --- | --- |

| PAIN AT ITS **WORST** (1) |  |
| --- | --- |
| PAIN AT ITS **LEAST** (2) |  |
| PAIN ON **AVERAGE** (3) |  |

End of Block: CHARACTERISTICS OF PAIN SYMPTOMS - PERIPHERAL NEUROPATHIC PAIN

Start of Block: CHARACTERISTICS OF PAIN SYMPTOMS - RLS PAIN

Q13.1 **SUB-SECTION A.8 : RESTLESS LEGS SYNDROME (RLS)**

 This sub-section asks about pain related to an **irresistible urge to move** that is often accompanied with a **burning, tingling, itching and/or throbbing sensation** in the **legs and/or arms** when you sit still or lie down.

 These symptoms usually occur during the evening and at night and are partially relieved or temporarily abolished with movement.

 We refer to this type of pain as RLS pain.

Q13.2 In the past month, have you experienced RLS pain?

- Yes (1)
- No (2)

Skip To: End of Block If Q13.2 = No

| Page Break |  |
| --- | --- |

Q13.3 Please indicate the area(s) of the body where your RLS pain occurs. (Select all that apply)

- Leg(s) (1)
- Arm(s) (2)

Q13.4 How long have you had RLS pain?

- Less than 3 months (8)
- 3 months to less than 1 year (9)
- More than 1 year (10)

Q13.5 Which of the following descriptions best describes your RLS pain in the past month?

- Continuous pain with slight variations (1)
- Continuous pain with large variations (2)
- Acute attacks of pain with pain free periods in-between (3)
- Acute attacks of pain on a background of constant pain (4)

Skip To: Q13.8 If Q13.5 = Continuous pain with slight variations

Skip To: Q13.8 If Q13.5 = Continuous pain with large variations

| Page Break |  |
| --- | --- |

Q13.6 On average, how often do you experience acute RLS pain attacks?

- Several times a day (1)
- Once a day (8)
- Two to six times a week (9)
- Once a week (10)
- Less than once a week (11)

Q13.7 On average, how long do your acute RLS pain attacks last?

- Seconds (1)
- Minutes (2)
- Hours (3)
- Days (4)

| Page Break |  |
| --- | --- |

Q13.8 Is your RLS pain **WORSE**... (Select all that apply)

- In the morning (3)
- At noon (4)
- In the afternoon (5)
- In the evening (6)
- At night (7)
- No difference (8)

Q13.9 Is your RLS pain **WORSE** during... (Select all that apply)

- **Peak-dose periods** when your Parkinson’s medication is most effective (30-90 mins after medication consumption) (1)
- **Wearing-off or off periods** when your Parkinson’s medication is not working (6)
- **Early-dose** **periods** (10-30 mins after medication consumption) (7)
- No difference (5)
- I don’t take Parkinson’s medication (8)

| Page Break |  |
| --- | --- |

Q13.10 What factors, if any, increase and/or trigger your RLS pain? (Select all that apply)

- Nothing/not sure (1)
- Eating (6)
- Speaking (7)
- Hot weather (8)
- Cold weather (9)
- Humid weather (10)
- No movement (13)
- Movement (14)
- Sleep or rest (15)
- Urination or defecation (17)
- Negative mood/stress (18)
- Bright lights (20)
- Loud noises (21)
- Exercise (24)
- Fatigue (25)
- Other (please specify) (26) __________________________________________________

| Page Break |  |
| --- | --- |

Q13.11 What factors, if any, decrease your RLS pain? (Select all that apply)

- Nothing/not sure (1)
- Eating (6)
- Speaking (7)
- Hot weather (8)
- Cold weather (9)
- Humid weather (10)
- No movement (12)
- Movement (13)
- Sleep or rest (14)
- Distraction (e.g. TV, reading, music) (16)
- Urination or defecation (17)
- Exercise (20)
- Other (please specify) (21) __________________________________________________

| Page Break |  |
| --- | --- |

Q13.12 Please rate your RLS pain by selecting the one number that best describes your pain in the past month: 

 **(0 = no pain : 10 = pain as bad as you can imagine)**

|  | 0 | 1 | 2 | 3 | 4 | 5 | 6 | 7 | 8 | 9 | 10 |
| --- | --- | --- | --- | --- | --- | --- | --- | --- | --- | --- | --- |

| PAIN AT ITS **WORST** (1) |  |
| --- | --- |
| PAIN AT ITS **LEAST** (2) |  |
| PAIN ON **AVERAGE** (3) |  |

End of Block: CHARACTERISTICS OF PAIN SYMPTOMS - RLS PAIN

Start of Block: CHARACTERISTICS OF PAIN SYMPTOMS - NOCTURNAL AKINESIA

Q14.1 **SUB-SECTION A.9 : NOCTURNAL AKINESIA**

 This sub-section asks about pain related to **difficulties when turning in bed at night** due to **rigidity/stiffness**.

 We refer to this type of pain as nocturnal akinesia.

Q14.2 In the past month, have you experienced nocturnal akinesia?

- Yes (1)
- No (2)

Skip To: End of Block If Q14.2 = No

| Page Break |  |
| --- | --- |

Q14.3 How long have you had nocturnal akinesia?

- Less than 3 months (8)
- 3 months to less than 1 year (9)
- More than 1 year (10)

Q14.4 On average, how often do you experience nocturnal akinesia?

- Every night (1)
- Two to six times a week (2)
- Once a week (3)
- Less than once a week (4)

Q14.5 Is your nocturnal akinesia **WORSE**... (Select all that apply)

- In the first third of the night (3)
- In the middle of the night (4)
- In the last third of the night (5)
- No difference (6)

Q14.6 Is your nocturnal akinesia **WORSE** during... (Select all that apply)

- **Peak-dose** **periods** when your Parkinson’s medication is most effective (30-90 mins after medication consumption) (1)
- **Wearing-off or off periods** when your Parkinson’s medication is not working (4)
- **Early-dose periods** (10-30 mins after medication consumption) (5)
- No difference (3)
- I don’t take Parkinson’s medication (6)

| Page Break |  |
| --- | --- |

Q14.7 What factors, if any, increase and/or trigger your nocturnal akinesia? (Select all that apply)

- Nothing/not sure (1)
- Eating (6)
- Speaking (7)
- Hot weather (8)
- Cold weather (9)
- Humid weather (10)
- No movement (13)
- Movement (14)
- Sleep or rest (15)
- Urination or defecation (17)
- Negative mood/stress (18)
- Bright lights (20)
- Loud noises (21)
- Exercise (24)
- Fatigue (25)
- Other (please specify) (26) __________________________________________________

| Page Break |  |
| --- | --- |

Q14.8 What factors, if any, decrease your nocturnal akinesia? (Select all that apply)

- Nothing/not sure (1)
- Eating (6)
- Speaking (7)
- Hot weather (8)
- Cold weather (9)
- Humid weather (10)
- No movement (12)
- Movement (13)
- Sleep or rest (14)
- Distraction (e.g. TV, reading, music) (16)
- Urination or defecation (17)
- Exercise (20)
- Other (please specify) (21) __________________________________________________

| Page Break |  |
| --- | --- |

Q14.9 Please rate your nocturnal akinesia by selecting the one number that best describes your pain in the past month: 

 **(0 = no pain : 10 = pain as bad as you can imagine)**

|  | 0 | 1 | 2 | 3 | 4 | 5 | 6 | 7 | 8 | 9 | 10 |
| --- | --- | --- | --- | --- | --- | --- | --- | --- | --- | --- | --- |

| PAIN AT ITS **WORST** (1) |  |
| --- | --- |
| PAIN AT ITS **LEAST** (2) |  |
| PAIN ON **AVERAGE** (3) |  |

End of Block: CHARACTERISTICS OF PAIN SYMPTOMS - NOCTURNAL AKINESIA

Start of Block: CHARACTERISTICS OF PAIN SYMPTOMS - CENTRAL PAIN

Q15.1 **SUB-SECTION A.10 : CENTRAL PARKINSON'S PAIN**

 This sub-section asks about central Parkinson’s pain.

 Central Parkinson’s pain is pain that is **not** explained by muscle rigidity/stiffness, movement, muscle cramps/dystonia, radicular and peripheral neuropathy, gastrointestinal dysfunction (e.g. constipation) or other non-Parkinson's syndromes/conditions.

 It is commonly described as **dull, vague, aching or cramp like sensation**, **severe itching**, **painful burning**, or **cutting/slicing**.

 This unusual type of pain may be **persistent** or **intermittent**, it may affect the **whole body**, or **one or more body parts** (e.g. face, abdomen or genitals), and in some instances, may move around the body. It is also usually **moderate to severe in intensity**.

Q15.2 In the past month, have you experienced central Parkinson’s pain?

- Yes (1)
- No (2)

Skip To: End of Block If Q15.2 = No

| Page Break |  |
| --- | --- |

Q15.3 Please indicate the area(s) of the body where your central Parkinson's pain occurs. (Select all that apply)

- Face (1)
- Head (2)
- Throat (3)
- Arm(s) (14)
- Upper abdomen (above the belly button) (4)
- Lower abdomen (below the belly button) (13)
- Rectum (6)
- Genitals (7)
- Leg(s) (15)
- Whole body (11)
- Other (please specify) (8) __________________________________________________

Q15.4 How long have you had central Parkinson's pain?

- Less than 3 months (8)
- 3 months to less than 1 year (9)
- More than 1 year (10)

Q15.5 Which of the following descriptions best describes your central Parkinson's pain in the past month?

- Continuous pain with slight variations (1)
- Continuous pain with large variations (2)
- Acute attacks of pain with pain free periods in-between (3)
- Acute attacks of pain on a background of constant pain (4)

Skip To: Q15.8 If Q15.5 = Continuous pain with slight variations

Skip To: Q15.8 If Q15.5 = Continuous pain with large variations

| Page Break |  |
| --- | --- |

Q15.6 On average, how often do you experience acute central Parkinson's pain attacks?

- Several times a day (1)
- Once a day (8)
- Two to six times a week (9)
- Once a week (10)
- Less than once a week (11)

Q15.7 On average, how long do your acute central Parkinson's pain attacks last?

- Seconds (1)
- Minutes (2)
- Hours (3)
- Days (4)

| Page Break |  |
| --- | --- |

Q15.8 Is your central Parkinson's pain **WORSE**... (Select all that apply)

- In the morning (3)
- At noon (4)
- In the afternoon (5)
- In the evening (6)
- At night (7)
- No difference (8)

Q15.9 Is your central Parkinson's pain **WORSE** during... (Select all that apply)

- **Peak-dose** **periods** when your Parkinson’s medication is most effective (30-90 mins after medication consumption) (1)
- **Wearing-off or off periods** when your Parkinson’s medication is not working (4)
- **Early-dose periods** (10-30 mins after medication consumption) (5)
- No difference (3)
- I don’t take Parkinson’s medication (6)

| Page Break |  |
| --- | --- |

Q15.10 What factors, if any, increase and/or trigger your central Parkinson's pain? (Select all that apply)

- Nothing/not sure (1)
- Eating (6)
- Speaking (7)
- Hot weather (8)
- Cold weather (9)
- Humid weather (10)
- No movement (13)
- Movement (14)
- Sleep or rest (15)
- Urination or defecation (17)
- Negative mood/stress (18)
- Bright lights (20)
- Loud noises (21)
- Exercise (24)
- Fatigue (25)
- Other (please specify) (26) __________________________________________________

| Page Break |  |
| --- | --- |

Q15.11 What factors, if any, decrease your central Parkinson's pain? (Select all that apply)

- Nothing/not sure (1)
- Eating (6)
- Speaking (7)
- Hot weather (8)
- Cold weather (9)
- Humid weather (10)
- No movement (12)
- Movement (13)
- Sleep or rest (14)
- Distraction (e.g. TV, reading, music) (16)
- Urination or defecation (17)
- Exercise (20)
- Other (please specify) (21) __________________________________________________

| Page Break |  |
| --- | --- |

Q15.12 Please rate your central Parkinson's pain by selecting the one number that best describes your pain in the past month: 

 **(0 = no pain : 10 = pain as bad as you can imagine)**

|  | 0 | 1 | 2 | 3 | 4 | 5 | 6 | 7 | 8 | 9 | 10 |
| --- | --- | --- | --- | --- | --- | --- | --- | --- | --- | --- | --- |

| PAIN AT ITS **WORST** (1) |  |
| --- | --- |
| PAIN AT ITS **LEAST** (2) |  |
| PAIN ON **AVERAGE** (3) |  |

End of Block: CHARACTERISTICS OF PAIN SYMPTOMS - CENTRAL PAIN

Start of Block: BREAK

Q16.1 You are now about half way through the survey. Thank you for your time and effort thus far.

 If you feel like you need a break, now might be a good time.

 Remember, you can return to the survey later by clicking on the survey link found on the **APPS website** (i.e., the same link you just before). This link will return you to the last question you answered in the survey. The answers to the questions you already provided will be automatically recorded/saved.

 Please note that if you would like to complete the survey over multiple sessions, **you will need to use the same device (i.e., the same computer or phone) and the same browser (i.e., Explorer, Safari, Chrome).**

End of Block: BREAK

Start of Block: INTRO TO COMMUNICATION WITH HCP

Q17.1
**SECTION B : COMMUNICATION  WITH HEALTHCARE PROFESSIONALS**
  
 The following section asks about your communication with healthcare professionals.

 **REMINDER: For this survey, the term 'Parkinson's related pain' refers to pain symptoms that are commonly experienced by people with Parkinson’s.**

 **These are the 10 pain symptoms that were included in SECTION A : CHARACTERISTICS OF PAIN SYMPTOMS.**

End of Block: INTRO TO COMMUNICATION WITH HCP

Start of Block: COMMUNICATION WITH NURSES

Q18.1 **SUB-SECTION B.1 : COMMUNICATION WITH NURSES**

 This sub-section asks about your communications with nurses.

Q18.2 Have you ever discussed your Parkinson's related pain with a nurse?

- Yes (9)
- No (10)
- Can't remember (11)

End of Block: COMMUNICATION WITH NURSES

Start of Block: COMMUNICATION WITH MEDICAL DOCTORS

Q19.1 **SUB-SECTION B.2 : COMMUNICATION WITH MEDICAL DOCTORS/SPECIALISTS**

 This sub-section asks about your communications with medical doctors/specialists.

 For this survey, the term ‘medical doctors/specialists’ refers to: **General practitioners (GPs)** **Specialists/physicians** (e.g. neurologists, geriatricians, psychiatrists, pain medicine specialists, anaesthetists) **Oral health professionals** (e.g. dentists)
 This does **not** include chiropractors, osteopaths, and other allied health professionals such as physiotherapists.

Q19.2 Have you ever discussed your Parkinson's related pain with a medical doctor/specialist?

- Yes (9)
- No (10)
- Can't remember (11)

Skip To: End of Block If Q19.2 = No

Skip To: End of Block If Q19.2 = Can't remember

Q19.3 Please indicate the medical doctor/specialist(s) you have discussed your Parkinson's related pain with? (Select all that apply)

- Neurologist (1)
- Geriatrician (3)
- General practitioner (GP) (4)
- Pain medicine specialist (5)
- Anaesthetist (12)
- Neurosurgeon (6)
- Sports and exercise physician (7)
- Orthopaedic surgeon (8)
- Psychiatrist (9)
- Dentist/orthodonist (10)
- Other medical doctor/specialist (please specify) (11) __________________________________________________

End of Block: COMMUNICATION WITH MEDICAL DOCTORS

Start of Block: COMMUNICATION WITH ALLIED HEALTH PROFESSIONAL

Q20.1 **SUB-SECTION B.3 : COMMUNICATION WITH ALLIED HEALTH PROFESSIONALS**

 This sub-section asks about your communications with allied health professionals.

 For this survey, the term ‘allied health professional’ refers to any non-medical health professional such as **exercise physiologists, physiotherapists, pharmacists, psychologists, speech pathologists, chiropractors, naturopaths, and massage therapists.**

Q20.2 Have you ever discussed your Parkinson's related pain with an allied health professional?

- Yes (1)
- No (2)
- Can't remember (3)

Skip To: End of Block If Q20.2 = No

Skip To: End of Block If Q20.2 = Can't remember

Q20.3 Please indicate the allied health professional(s) you have discussed your Parkinson's related pain with? (Select all that apply)

- Psychologist/neuropsychologist (1)
- Social worker/counsellor (2)
- Physiotherapist/neurophysiotherapist (3)
- Podiatrist (4)
- Dietician (5)
- Chiropractor (6)
- Osteopath (14)
- Exercise physiologist (7)
- Occupational therapist (8)
- Speech pathologist (9)
- Massage therapist (10)
- Naturopath (11)
- Pharmacist (12)
- Other allied health professional (please specify) (13) __________________________________________________

End of Block: COMMUNICATION WITH ALLIED HEALTH PROFESSIONAL

Start of Block: INTRO TO TREATMENT PRACTICES

Q21.1 **SECTION C : PAIN TREATMENT PRACTICES**

 The following section asks about your use of therapies, including prescription and non-prescription **medications**, to manage **Parkinson’s related pain.**

 To make it easier for you to complete these sections, it may be useful to have your **medications on hand for reference**.

 **REMINDER: For this survey, the term 'Parkinson's related pain' refers to pain symptoms that are commonly experienced by people with Parkinson’s.**

 **These are the 10 pain symptoms that were included in SECTION A : CHARACTERISTICS OF PAIN SYMPTOMS.**

End of Block: INTRO TO TREATMENT PRACTICES

Start of Block: PRESCRIPTION OPIOIDS

Q22.1 **SUB-SECTION C.1 : PRESCRIPTION OPIOIDS**

 This sub-section asks about the use of oral (e.g. tablets, capsules) and transdermal (e.g. patches) **prescription opioids** such as **oxycodone (e.g. Targin, OxyContin, Endone), fentanyl (e.g., Durogesic), tramadol, and codeine (e.g. Panadeine Forte)**.

 This does not include the use of prescription paracetamol, aspirin, and ibuprofen where these medications are the only active ingredients.

Q22.2 Have you ever used prescription opioids for Parkinson's related pain?

- Yes (1)
- No (2)

Skip To: End of Block If Q22.2 = No

| Page Break |  |
| --- | --- |

Q22.3 In the past month, have you used prescription opioids for Parkinson's related pain?

- Yes (1)
- No (2)

Skip To: Q22.7 If Q22.3 = No

| Page Break |  |
| --- | --- |

Q22.4 Please indicate the prescription opioids you have used in the past month for Parkinson's related pain. (Select all that apply)

- Oxycodone + naloxone (e.g. Targin) (1)
- Oxycodone (e.g. OxyNorm, OxyContin, Endone) (2)
- Paracetamol + codeine (e.g. Panadeine Forte, Panamax Co) (3)
- Aspirin + codeine (e.g. Aspalgin) (4)
- Tramadol (e.g. Tramal, Zydol) (5)
- Tapentadol (e.g. Palexia) (6)
- Fentanyl (e.g. Durogesic Patch) (7)
- Buprenorphine (e.g. Norspan Patch) (8)
- Other prescription opioids (please specify) (9) __________________________________________________

Q22.5 In the past month, how often did you use prescription opioids for Parkinson's related pain?

- Every day (1)
- Two to six times a week (6)
- Once a week (2)
- Less than once a week (3)

Q22.6 In the past month, how effective, if at all, have prescription opioids been in reducing your Parkinson's related pain?

- Not effective at all (1)
- Slightly effective (2)
- Moderately effective (3)
- Very effective (4)
- Extremely effective (5)

| Page Break |  |
| --- | --- |

Q22.7 Where did/do you usually obtain the prescription for the opioids you use for Parkinson's related pain?

- Neurologist (1)
- Geriatrician (3)
- General practitioner (GP) (4)
- Pain medicine specialist (5)
- Anaesthetist (12)
- Neurosurgeon (6)
- Nurse practitioner (7)
- Sports and exercise physician (8)
- Orthopaedic surgeon (9)
- Psychiatrist (10)
- Other medical doctor/specialist (please specify) (11) __________________________________________________

End of Block: PRESCRIPTION OPIOIDS

Start of Block: PRESCRIPTION ANTICONVULSANTS

Q23.1 **SUB-SECTION C.2 : PRESCRIPTION ANTICONVULSANTS**

 This sub-section asks about the use of oral (e.g. tablets, capsules) **prescription anticonvulsants** such as **pregabalin (e.g. Lyrica) and gabapentin (e.g. Neurontin)**.

Q23.2 Have you ever used prescription anticonvulsants for Parkinson's related pain?

- Yes (1)
- No (2)

Skip To: End of Block If Q23.2 = No

| Page Break |  |
| --- | --- |

Q23.3 In the past month, have you used prescription anticonvulsants for Parkinson's related pain?

- Yes (1)
- No (2)

Skip To: Q23.6 If Q23.3 = No

| Page Break |  |
| --- | --- |

Q23.4 Please indicate the prescription anticonvulsants you have used in the past month for Parkinson's related pain. (Select all that apply)

- Pregabalin (e.g. Lyrica, Lypralin, Lyzalon, Neuroccord) (1)
- Gabapentin (e.g. Neurontin, Gabacor, Nupentin) (2)
- Carbamazepine (e.g. Tegretol) (3)
- Other prescription anticonvulsants (please specify) (4) __________________________________________________

Q23.5 In the past month, how effective, if at all, have prescription anticonvulsants been in reducing your Parkinson's related pain?

- Not effective at all (1)
- Slightly effective (2)
- Moderately effective (3)
- Very effective (4)
- Extremely effective (5)

Q23.6 Where did/do you usually obtain the prescription for the anticonvulsants you use for Parkinson's related pain?

- Neurologist (1)
- Geriatrician (3)
- General practitioner (GP) (4)
- Pain medicine specialist (5)
- Anaesthetist (12)
- Neurosurgeon (6)
- Nurse practitioner (7)
- Sports and exercise physician (8)
- Orthopaedic surgeon (9)
- Psychiatrist (10)
- Other medical doctor/specialist (please specify) (11) __________________________________________________

End of Block: PRESCRIPTION ANTICONVULSANTS

Start of Block: PRESCRIPTION NSAIDS

Q24.1 **SUB-SECTION C.3 : PRESCRIPTION NONSTEROIDAL ANTI-INFLAMMATORY DRUGS**

 This sub-section asks about the use of oral (e.g. tablets, capsules) and rectal (e.g. suppositories) **prescription nonsteroidal anti-inflammatory drugs** such as **Celebrex, Voltaren, Indocid, and Naprosyn**.

 This does not include creams/ointments/gels.
  
NOTE: For this survey, the term 'prescription nonsteroidal anti-inflammatory drugs' refers to nonsteroidal anti-inflammatory medications that have been obtained with a script/prescription from an authorised health professional (e.g. medical doctor/specialist or nurse practitioner).

Q24.2 Have you ever used prescription nonsteroidal anti-inflammatory drugs for Parkinson's related pain?

- Yes (1)
- No (2)

Skip To: End of Block If Q24.2 = No

| Page Break |  |
| --- | --- |

Q24.3 In the past month, have you used prescription nonsteroidal anti-inflammatory drugs for Parkinson's related pain?

- Yes (1)
- No (2)

Skip To: Q24.7 If Q24.3 = No

| Page Break |  |
| --- | --- |

Q24.4 Please indicate the prescription nonsteroidal anti-inflammatory drugs you have used in the past month for Parkinson's related pain. (Select all that apply)

- Celecoxib (e.g. Celebrex) (1)
- Diclofenac (e.g. Voltaren, Voltaren Suppositories, Fenac, Clonac) (2)
- Meloxicam (e.g. Mobic, Movalis, Moxicam, Melobic) (3)
- Naproxen (e.g. Naprosyn, Proxen) (4)
- Indomethacin (e.g. Indocid, Indocid Suppositories, Arthrexin) (6)
- Other prescription nonsteroidal anti-inflammatory drugs (please specify) (5) __________________________________________________

Q24.5 In the past month, how often did you use prescription nonsteroidal anti-inflammatory drugs for Parkinson's related pain?

- Every day (1)
- Two to six times a week (6)
- Once a week (7)
- Less than once a week (8)

Q24.6 In the past month, how effective, if at all, have prescription nonsteroidal anti-inflammatory drugs been in reducing your Parkinson's related pain?

- Not effective at all (1)
- Slightly effective (2)
- Moderately effective (3)
- Very effective (4)
- Extremely effective (5)

| Page Break |  |
| --- | --- |

Q24.7 Where did/do you usually obtain the prescription for your nonsteroidal anti-inflammatory drugs you use for Parkinson's related pain?

- Neurologist (1)
- Geriatrician (3)
- General practitioner (GP) (4)
- Pain medicine specialist (5)
- Anaesthetist (12)
- Neurosurgeon (6)
- Nurse practitioner (7)
- Sports and exercise physician (8)
- Orthopaedic surgeon (9)
- Psychiatrist (10)
- Other medical doctor/specialist (please specify) (11) __________________________________________________

End of Block: PRESCRIPTION NSAIDS

Start of Block: PRESCRIPTION ANTIDEPRESSANTS

Q25.1 **SUB-SECTION C.4 : PRESCRIPTION ANTIDEPRESSANTS**

 This sub-section asks about the use of oral (e.g. tablets, capsules) **prescription antidepressants** such as **amitriptyline (e.g. Endep), nortriptyline (e.g. Allegron), and SNRIs (e.g. duloxetine and venlafaxine)**.

Q25.2 Have you ever used prescripion antidepressants for Parkinson's related pain?

- Yes (1)
- No (2)

Skip To: End of Block If Q25.2 = No

| Page Break |  |
| --- | --- |

Q25.3 In the past month, have you used prescription antidepressants for Parkinson's related pain?

- Yes (1)
- No (2)

Skip To: Q25.6 If Q25.3 = No

| Page Break |  |
| --- | --- |

Q25.4 Please indicate the prescription antidepressants you have used in the past month for Parkinson's related pain. (Select all that apply)

- Amitriptyline (e.g. Endep, Entrip) (1)
- Nortriptyline (e.g. NortriTABS, Allegron) (2)
- Duloxetine (e.g. Cymbalta, Tixol, Dytrex) (3)
- Venlafaxine (e.g. Efexor, Elaxine, Enlafax) (4)
- Mirtazapine (e.g. Avanza, Mirtanza, Axit) (6)
- Other prescription antidepressants (please specify) (5) __________________________________________________

Q25.5 In the past month, how effective, if at all, have prescription antidepressants been in reducing your Parkinson's related pain?

- Not effective at all (1)
- Slightly effective (2)
- Moderately effective (3)
- Very effective (4)
- Extremely effective (5)

Q25.6 Where did/do you usually obtain the prescription for the antidepressants you use for Parkinson's related pain?

- Neurologist (1)
- Geriatrician (3)
- General practitioner (GP) (4)
- Pain medicine specialist (5)
- Anaesthetist (12)
- Neurosurgeon (6)
- Nurse practitioner (7)
- Sports and exercise physician (8)
- Orthopaedic surgeon (9)
- Psychiatrist (10)
- Other medical doctor/specialist (please specify) (11) __________________________________________________

End of Block: PRESCRIPTION ANTIDEPRESSANTS

Start of Block: MEDICINAL CANNABIS

Q26.1 **SUB-SECTION C.5 : MEDICINAL CANNABIS**

 This sub-section asks about the use of **medicinal cannabis**.

 NOTE: For this survey, the term 'medicinal cannabis' refers to cannabis products that have been obtained with a script/prescription from an authorised health professional (e.g. medical doctor/specialist or nurse practitioner).

Q26.2 Have you ever used medicinal cannabis for Parkinson's related pain?

- Yes (1)
- No (2)

Skip To: End of Block If Q26.2 = No

| Page Break |  |
| --- | --- |

Q26.3 In the past month, have you used medicinal cannabis for Parkinson's related pain?

- Yes (1)
- No (2)

Skip To: Q26.6 If Q26.3 = No

| Page Break |  |
| --- | --- |

Q26.4 In the past month, how often did you use medicinal cannabis for Parkinson's related pain?

- Every day (1)
- Two to six times a week (6)
- Once a week (7)
- Less than once a week (8)

Q26.5 In the past month, how effective, if at all, has medicinal cannabis been in reducing your Parkinson's related pain?

- Not effective at all (1)
- Slightly effective (2)
- Moderately effective (3)
- Very effective (4)
- Extremely effective (5)

Q26.6 Where did/do you usually obtain the prescription for the medicinal cannabis you use for Parkinson's related pain?

- Neurologist (1)
- Geriatrician (3)
- General practitioner (GP) (4)
- Pain medicine specialist (5)
- Anaesthetist (12)
- Neurosurgeon (6)
- Nurse practitioner (7)
- Sports and exercise physician (8)
- Orthopaedic surgeon (9)
- Psychiatrist (10)
- Other medical doctor/specialist (please specify) (11) __________________________________________________

End of Block: MEDICINAL CANNABIS

Start of Block: PRESCRIPTION TOPICAL ANALGESICS

Q27.1 **SUB-SECTION C.6 : PRESCRIPTION TOPICAL ANALGESICS**
  
This sub-section asks about the use of **prescription topical analgesics**. 
 
NOTE: For this survey, the term ‘prescription topical analgesics’ refers to any **creams, ointments or gels used for the management of pain** that have been obtained with a script/prescription from an authorised health professional (e.g. medical doctor/specialist) that are often dispensed by a compounding pharmacist.

Q27.2 Have you ever used prescription topical analgesics for Parkinson's related pain?

- Yes (1)
- No (2)

Skip To: End of Block If Q27.2 = No

| Page Break |  |
| --- | --- |

Q27.3 In the past month, have you used prescription topical analgesics for Parkinson's related pain?

- Yes (1)
- No (2)

Skip To: Q27.6 If Q27.3 = No

| Page Break |  |
| --- | --- |

Q27.4 In the past month, how often did you use prescription topical analgesics for Parkinson's related pain?

- Every day (1)
- Two to six times a week (6)
- Once a week (7)
- Less than once a week (8)

Q27.5 In the past month, how effective, if at all, have prescription topical analgesics been in reducing your Parkinson's related pain?

- Not effective at all (1)
- Slightly effective (2)
- Moderately effective (3)
- Very effective (4)
- Extremely effective (5)

Q27.6 Where did/do you usually obtain the prescription for your topical analgesics you use for Parkinson's related pain?

- Neurologist (1)
- Geriatrician (3)
- General practitioner (GP) (4)
- Pain medicine specialist (5)
- Anaesthetist (12)
- Neurosurgeon (6)
- Nurse practitioner (7)
- Sports and exercise physician (8)
- Orthopaedic surgeon (9)
- Psychiatrist (10)
- Other medical doctor/specialist (please specify) (11) __________________________________________________

End of Block: PRESCRIPTION TOPICAL ANALGESICS

Start of Block: NON-PRESCRIPTION PAIN MEDICATION

Q28.1 **SUB-SECTION C.7 : NON-PRESCRIPTION PAIN MEDICATION**

 This sub-section asks about the use of oral (e.g. tablets, capsules) **non-prescription pain medication** (e.g. over-the-counter drugs) such as **paracetamol (e.g. Panadol) and non-steroidal anti-inflammatory drugs (e.g. Nurofen, Voltaren, Aspro Clear)**.

 This does not include creams/ointments/gels.

Q28.2 Have you ever used non-prescription pain medication for Parkinson's related pain?

- Yes (1)
- No (2)

Skip To: End of Block If Q28.2 = No

| Page Break |  |
| --- | --- |

Q28.3 In the past month, have you used non-prescription pain medication for Parkinson's related pain?

- Yes (1)
- No (2)

Skip To: End of Block If Q28.3 = No

| Page Break |  |
| --- | --- |

Q28.4 Please indicate the non-prescription pain medication(s) you have used in the past month for Parkinson's related pain. (Select all that apply)

- Paracetamol (e.g. Panadol, Panamax) (1)
- Ibuprofen (e.g. Nurofen, Advil) (2)
- Paracetamol + ibuprofen (e.g. Nuromol, Maxigensic, Mersynofen) (3)
- Diclofenac (e.g. Voltaren) (4)
- Naproxen (e.g. Naprogesic) (5)
- Aspirin (e.g. Aspro Clear) (6)
- Other non-prescription pain medication (please specify) (7) __________________________________________________

Q28.5 In the past month, how often did you use non-prescription pain medication for Parkinson's related pain?

- Every day (1)
- Two to six times a week (6)
- Once a week (7)
- Less than once a week (8)

Q28.6 In the past month, how effective, if at all, has non-prescription pain medication been in reducing your Parkinson's related pain?

- Not effective at all (1)
- Slightly effective (2)
- Moderately effective (3)
- Very effective (4)
- Extremely effective (5)

End of Block: NON-PRESCRIPTION PAIN MEDICATION

Start of Block: NON-PRESCRIPTION TOPICAL ANALGESICS

Q29.1 **SUB-SECTION C.8 : NON-PRESCRIPTION TOPICAL ANALGESICS**

 This sub-section asks about the use of **non-prescription topical analgesics**.

 For this survey, the term ‘non-prescription topical analgesics’ refers to any **creams, gels and patches used for the management of pain** that can be obtained without a prescription (OTC), such as **ibuprofen (e.g. Voltaren), diclofenac (Voltaren Emulgel), and capsaicin (e.g. Zostrix)**.

Q29.2 Have you ever used non-prescription topical analgesics for Parkinson's related pain?

- Yes (1)
- No (2)

Skip To: End of Block If Q29.2 = No

| Page Break |  |
| --- | --- |

Q29.3 In the past month, have you used non-prescription topical analgesics for Parkinson's related pain?

- Yes (1)
- No (2)

Skip To: End of Block If Q29.3 = No

| Page Break |  |
| --- | --- |

Q29.4 Please indicate the non-prescription topical analgesics you have used in the past month for Parkinson's related pain. (Select all that apply)

- Diclofenac Gel (e.g. Voltaren Emulgel) (1)
- Ibuprofen Gel (e.g. Nurofen) (2)
- Benzydamine Gel (e.g. Difflam) (3)
- Capsaicin Cream (e.g. Zostrix) (4)
- Piroxicam Gel (e.g. Feldene Gel) (5)
- Other non-prescription topical analgesics (please specify) (6) __________________________________________________

Q29.5 In the past month, how often did you use non-prescription topical analgesics for Parkinson's related pain?

- Every day (1)
- Two to six times a week (6)
- Once a week (7)
- Less than once a week (8)

Q29.6 In the past month, how effective, if at all, have non-prescription topical analgesics been in reducing your Parkinson's related pain?

- Not effective at all (1)
- Slightly effective (2)
- Moderately effective (3)
- Very effective (4)
- Extremely effective (5)

End of Block: NON-PRESCRIPTION TOPICAL ANALGESICS

Start of Block: PARKINSON'S MEDICATION

Q30.1 **SUB-SECTION C.9 : PRESCRIPTION PARKINSON’S MEDICATION**

 This sub-section asks about the use of oral (e.g. tablets, capsules), enteral (e.g. intestinal gels), transdermal (e.g. patches), and subcutaneous (e.g. injections) **prescription Parkinson’s medication** such as: **Levodopa (e.g. Sinemet, Duodopa, Madopar, Stalevo)** **Dopamine agonists (e.g. Sifrol, Cabaser, Parlodel, Neupro, Movapa)** **COMT inhibitors (e.g. Comtan)**  **MOA type B inhibitors (e.g. Eldepryl, Azilect, Xadago)**

Q30.2 Have you used prescription Parkinson's medication in the past month?

- Yes (1)
- No (2)

Skip To: End of Block If Q30.2 = No

| Page Break |  |
| --- | --- |

Q30.3 Please indicate the prescription Parkinson's medication(s) you have used in the past month. (Select all that apply)

- **Dopa and dopa derivatives – for example** levodopa + carbidopa (e.g. Sinemet, Kinson, Duodopa Intestinal Gel) levodopa + benserazide (e.g. Madopar) levodopa + carbidopa + entacapone (e.g. Stalevo, Carlevent, Lecteva) (1)
- **Adamantane derivatives – for example** amantadine (e.g. Symmetrel) (2)
- **Dopamine agonists – for example** pramipexole (e.g. Sifrol, Simipex, Simpral) cabergoline (e.g. Cabaser, Dostinex) bromocriptine (e.g. Parlodel) rotigotine (e.g. Neupro Patch) apomorphine ( e.g. Movapa Injection) (3)
- **COMT inhibitors – for example** entacapone (brand name(s): Comtan) opicapone (brand nam(s): Ongentys) (4)
- **Irreversible MAO type B inhibitors – for example** selegiline (brand name(s): Eldepryl) rasagiline (brand name(s): Azilect, Alziras) (5)
- **Reversible MAO type B inhibitors – for example** safinamide (brand name(s): Xadago) (6)
- **Anticholinergic agents – for example** trihexyphenidyl (brand name(s): Artane) benzatrophine (brand name(s): Benztrop, Benztrophine Injection) (7)
- Other prescription Parkinson's medication (please specify) (8) __________________________________________________

| Page Break |  |
| --- | --- |

Q30.4 In the past month, how effective, if at all, has your prescription Parkinson's medication been in reducing Parkinson's related pain?

- Not effective at all (1)
- Slightly effective (2)
- Moderately effective (3)
- Very effective (4)
- Extremely effective (5)

Q30.5 Where do you usually obtain the prescription for your Parkinson's medication?

- Neurologist (1)
- Geriatrician (3)
- General practitioner (GP) (4)
- Other medical doctor/specialist (please specify) (5) __________________________________________________

End of Block: PARKINSON'S MEDICATION

Start of Block: EXERCISE THERAPY

Q31.1 **SUB-SECTION C.10 : EXERCISE THERAPY**

 This sub-section asks about **exercise therapy**.

 In this survey, the term ‘exercise therapy’ refers to any **structured** or **planned** physical activity such as **walking, jogging, aquatics, cycling, going to the gym, weight training, flexibility training, gait and balance training, Pilates and dance**.

 This does not include Tai Chi and Qi Gong.

Q31.2 Since you have been diagnosed with Parkinson’s, have you ever used exercise therapy?

- Yes (1)
- No (2)

Skip To: End of Block If Q31.2 = No

| Page Break |  |
| --- | --- |

Q31.3 Have you used exercise therapy in the past month?

- Yes (1)
- No (2)

Skip To: End of Block If Q31.3 = No

| Page Break |  |
| --- | --- |

Q31.4 Please indicate the form(s) of exercise therapy you have used in the past month. (Select all that apply)

- **Aqua-based training** (e.g. aquatic tai chi, aquatic exercise training, Halliwick aquatic exercises, water-based physiotherapy) (1)
- **Dance** (e.g. dance therapy, Ronnie Gardiner rhythm and music method, tango, waltz/foxtrot) (2)
- **Endurance training** (e.g. aerobic training, brisk walking, high-cadence cycling, Nordic walking, speed treadmill training) (3)
- **Flexibility training** (e.g. flexibility exercises, stretching, yoga) (4)
- **Gait/balance/functional training** (e.g. functional mobility training, HiBalance training, treadmill training) (5)
- **Gaming** (e.g. Exergaming, Nintendo Wii training) (6)
- **LSVT BIG** (7)
- **Strength/resistance training** (e.g. muscle power training, progressive resistance exercise) (8)
- Other exercise therapy (please specify) (9) __________________________________________________

| Page Break |  |
| --- | --- |

Q31.5 In the past month, on average, how many minutes per week of exercise therapy have you completed?

- Less than 60 minutes (1)
- 60 to less than 120 minutes (2)
- 120 to less than 180 minutes (3)
- 180 to less than 240 minutes (4)
- 240 to less than 300 minutes (5)
- 300 or more (6)

Q31.6 In the past month, how effective, if at all, has exercise therapy been in reducing your Parkinson's related pain?

- Not effective at all (1)
- Slightly effective (2)
- Moderately effective (3)
- Very effective (4)
- Extremely effective (5)

Q31.7 In the past month, did you use an exercise therapy trainer/instructor? For example, a personal trainer, gym/Pilates/yoga/dance instructor, exercise physiologist, occupational therapist or physiotherapist.

- Yes (1)
- No (2)

Skip To: End of Block If Q31.7 = No

| Page Break |  |
| --- | --- |

Q31.8 Please indicate the exercise therapy trainer(s)/instructor(s) you have used in the past month? (Select all that apply)

- Physiotherapist (1)
- Exercise physiologist (2)
- Occupational therapist (3)
- Personal trainer (4)
- Gym instructor (5)
- Pilates instructor (6)
- Yoga instructor (7)
- Dance instructor (8)
- Other exercise therapy trainer/instructor (please specify) (9) __________________________________________________

End of Block: EXERCISE THERAPY

Start of Block: MANIPULATIVE AND BODY-BASED THERAPY

Q32.1 **SUB-SECTION C.11 : MANIPULATIVE AND BODY-BASED THERAPY**

 This sub-section asks about **manipulative and body-based therapy**.

 In this survey, the term ‘manipulative and body-based therapy’ refers to practices that involve manipulation or movement of one or more parts of the body, such as **manual physical therapy (e.g. hands on treatment provided by a physiotherapist), spinal manipulation (e.g. chiropractic and osteopathic manipulation), massage, and reflexology**.

Q32.2 Have you ever used manipulative and body-based therapy for Parkinson's related pain?

- Yes (1)
- No (2)

Skip To: End of Block If Q32.2 = No

| Page Break |  |
| --- | --- |

Q32.3 In the past **3 months**, have you used manipulative body-based therapy for Parkinson's related pain?

- Yes (1)
- No (2)

Skip To: End of Block If Q32.3 = No

| Page Break |  |
| --- | --- |

Q32.4 Please indicate the allied health professional(s) you have used in the past **3 months** for manipulative and body-based therapy to treat Parkinson's related pain. (Select all that apply)

- Physiotherapist (1)
- Chiropractor (2)
- Osteopath (3)
- Massage therapist (4)
- Reflexologist (5)
- Other allied health professional (please specify) (6) __________________________________________________

Q32.5 In the past **3 months**, how often have you used manipulative and body-based therapy for Parkinson's related pain?

- Every day (1)
- Once a week or more (6)
- About once or twice a month (7)
- Every few months (8)

Q32.6 In the past **3 months**, how effective, if at all, has manipulative and body-based therapy been in reducing your Parkinson's related pain?

- Not effective at all (1)
- Slightly effective (2)
- Moderately effective (3)
- Very effective (4)
- Extremely effective (5)

End of Block: MANIPULATIVE AND BODY-BASED THERAPY

Start of Block: ELECTRICAL THERAPY

Q33.1 **SUB-SECTION C.12 : ELECTRICAL THERAPY**

 This sub-section asks about **electrical therapy**.

 In this survey, the term ‘electrical therapy’ refers to devices that deliver electrical stimulation to nerves such as **cranial electrotherapy stimulation (CES), electrotherapy/TENS (transcutaneous electrical nerve stimulation), and Tremor’s glove**.

 This does not include electrical acupuncture.

Q33.2 Have you ever used electrical therapy for Parkinson's related pain?

- Yes (1)
- No (2)

Skip To: End of Block If Q33.2 = No

| Page Break |  |
| --- | --- |

Q33.3 In the past **3 months**, have you used electrical therapy for Parkinson's related pain?

- Yes (1)
- No (2)

Skip To: End of Block If Q33.3 = No

| Page Break |  |
| --- | --- |

Q33.4 Please indicate the form(s) of electrical therapy you have used in the past **3 months** for Parkinson's related pain. (Select all that apply)

- Cranial electrotherapy stimulation (CES) (1)
- Electrotherapy/TENS (transcutaneous electrical nerve stimulation) (2)
- Tremor’s glove (3)
- Other electrical therapy (please specify) (4) __________________________________________________

Q33.5 In the past **3 months**, how often have you used electrical therapy for Parkinson's related pain?

- Every day (1)
- Once a week or more (6)
- About once or twice a month (7)
- Every few months (8)

Q33.6 In the past **3 months**, how effective, if at all, has electrical therapy been in reducing your Parkinson's related pain?

- Not effective at all (1)
- Slightly effective (2)
- Moderately effective (3)
- Very effective (4)
- Extremely effective (5)

End of Block: ELECTRICAL THERAPY

Start of Block: VIBRATION THERAPY

Q34.1 **SUB-SECTION C.13 : VIBRATION THERAPY**

 This sub-section asks about **vibration therapy**.

 In this survey, the term ‘vibration therapy’ refers to devices and procedures that deliver mechanical stimulation to muscles, tendons, and bones, such as **therapeutic ultrasound and vibration therapy (local or whole body)**.

Q34.2 Have you ever used vibration therapy for Parkinson's related pain?

- Yes (1)
- No (2)

Skip To: End of Block If Q34.2 = No

| Page Break |  |
| --- | --- |

Q34.3 In the past **3 months**, have you used vibration therapy for Parkinson's related pain?

- Yes (1)
- No (2)

Skip To: End of Block If Q34.3 = No

| Page Break |  |
| --- | --- |

Q34.4 Please indicate the form(s) of vibration therapy you have used in the past **3 months** for Parkinson's related pain. (Select all that apply)

- Therapeutic ultrasound (1)
- Vibration therapy (local or whole body) (2)
- Other vibration therapy (please specify) (3) __________________________________________________

Q34.5 In the past **3 months**, how often have you used vibration therapy for Parkinson's related pain?

- Every day (1)
- Once a week or more (6)
- About once or twice a month (7)
- Every few months (8)

Q34.6 In the past **3 months**, how effective, if at all, has vibration therapy been in reducing your Parkinson's related pain?

- Not effective at all (1)
- Slightly effective (2)
- Moderately effective (3)
- Very effective (4)
- Extremely effective (5)

End of Block: VIBRATION THERAPY

Start of Block: ACUPUNCTURE THERAPY

Q35.1 **SUB-SECTION C.14 : ACUPUNCTURE THERAPY**

 This sub-section asks about **acupuncture therapy**.

 In this survey, the term ‘acupuncture therapy’ refers to **traditional and electrical acupuncture**.

 This does not include dry needling.

Q35.2 Have you ever used acupuncture therapy for Parkinson's related pain?

- Yes (1)
- No (2)

Skip To: End of Block If Q35.2 = No

| Page Break |  |
| --- | --- |

Q35.3 In the past **3 months**, have you used acupuncture therapy for Parkinson's related pain?

- Yes (1)
- No (2)

Skip To: End of Block If Q35.3 = No

| Page Break |  |
| --- | --- |

Q35.4 Please indicate the form(s) of acupuncture therapy you have used in the past **3 months** for Parkinson's related pain. (Select all that apply)

- Traditional acupuncture (1)
- Electrical acupuncture (2)

Q35.5 In the past **3 months**, how often have you used acupuncture therapy for Parkinson's related pain?

- Every day (2)
- Once a week or more (6)
- About once or twice a month (7)
- Every few months (8)

Q35.6 In the past **3 months**, how effective, if at all, has acupuncture therapy been in reducing your Parkinson's related pain?

- Not effective at all (1)
- Slightly effective (2)
- Moderately effective (3)
- Very effective (4)
- Extremely effective (5)

End of Block: ACUPUNCTURE THERAPY

Start of Block: DRY NEEDLING THERAPY

Q36.1 **SUB-SECTION C.15 : DRY NEEDLING THERAPY**

 This sub-section asks about **dry needling therapy**.

Q36.2 Have you ever used dry needling therapy for Parkinson's related pain?

- Yes (1)
- No (2)

Skip To: End of Block If Q36.2 = No

| Page Break |  |
| --- | --- |

Q36.3 In the past **3 months**, have you used dry needling therapy for Parkinson's related pain?

- Yes (1)
- No (2)

Skip To: End of Block If Q36.3 = No

| Page Break |  |
| --- | --- |

Q36.4 In the past **3 months**, how often have you used dry needling therapy for Parkinson's related pain?

- Every day (1)
- Once a week or more (6)
- About once or twice a month (7)
- Every few months (2)

Q36.5 In the past **3 months**, how effective, if at all, has dry needling therapy been in reducing your Parkinson's related pain?

- Not effective at all (1)
- Slightly effective (2)
- Moderately effective (3)
- Very effective (4)
- Extremely effective (5)

End of Block: DRY NEEDLING THERAPY

Start of Block: BOTOX THERAPY

Q37.1 **SUB-SECTION C.16 : BOTULINUM TOXIN (BOTOX) THERAPY**

 This sub-section asks about **botulinum toxin (BOTOX) therapy**.

Q37.2 Have you ever used BOTOX therapy for Parkinson's related pain?

- Yes (1)
- No (2)

Skip To: End of Block If Q37.2 = No

Q37.3 In the past **12 months**, have you used BOTOX therapy for Parkinson's related pain?

- Yes (1)
- No (2)

Skip To: End of Block If Q37.3 = No

| Page Break |  |
| --- | --- |

Q37.4 In the past**12 months**, how often have you used BOTOX therapy for Parkinson's related pain?

- About once a month (1)
- Every few months (2)
- Once or twice a year (3)

Q37.5 In the past**12 months**, how effective, if at all, has BOTOX therapy been in reducing your Parkinson's related pain?

- Not effective at all (1)
- Slightly effective (2)
- Moderately effective (3)
- Very effective (4)
- Extremely effective (5)

End of Block: BOTOX THERAPY

Start of Block: MENTAL HEALTH THERAPY

Q38.1 **SUB-SECTION C.17 : MENTAL HEALTH THERAPY**

 This sub-section asks about **mental health therapy**.

 In this survey, the term ‘mental health therapy’ refers to **psychotherapy and counselling** services provided by psychiatrists, psychologists and social works/counsellors.

Q38.2 Have you ever used mental health therapy for Parkinson's related pain?

- Yes (1)
- No (2)

Skip To: End of Block If Q38.2 = No

| Page Break |  |
| --- | --- |

Q38.3 In the past **3 months**, have you used mental health therapy for Parkinson's related pain?

- Yes (1)
- No (2)

Skip To: End of Block If Q38.3 = No

| Page Break |  |
| --- | --- |

Q38.4 Please indicate the mental health therapist(s) you have used in the past **3 months** to treat Parkinson's related pain. (Select all that apply)

- Psychiatrist (1)
- Psychologist/neuropsychologist (2)
- Social worker/counsellor (3)
- Other mental health therapist (please specify) (4) __________________________________________________

Q38.5 In the past **3 months**, how often have you used mental health therapy for Parkinson's related pain?

- Every day (1)
- Once a week or more (5)
- About once or twice a month (6)
- Every few months (7)

Q38.6 In the past **3 months**, how effective, if at all, has mental health therapy therapy been in reducing your Parkinson's related pain?

- Not effective at all (1)
- Slightly effective (2)
- Moderately effective (3)
- Very effective (4)
- Extremely effective (5)

End of Block: MENTAL HEALTH THERAPY

Start of Block: PERCUTANEOUS SURGICAL THERAPY

Q39.1 **SUB-SECTION C.18 : PERCUTANEOUS SURGICAL THERAPY**

 This sub-section asks about **percutaneous surgical therapy**.

 In this survey, the term ‘percutaneous surgical therapy’ refers to **non-invasive procedures** typically performed in a day/outpatient surgery setting by a medical doctor/specialist using needles, such as **PENS (percutaneous electrical nerve stimulation)** **PNT (percutaneous neuromodulation therapy)** **Neurotomy (e.g. radiofrequency ablation, chemical ablation, rhizotomy)** **Nerve blockade (e.g. injection of anaesthetic or steroid to a nerve)** **Joint blockade (e.g. injection of anaesthetic or steroid to a joint)**

Q39.2 Have you ever used percutaneous surgical therapy for Parkinson's related pain?

- Yes (1)
- No (2)

Skip To: End of Block If Q39.2 = No

| Page Break |  |
| --- | --- |

Q39.3 In the past **12 months**, have you used percutaneous surgical therapy for Parkinson's related pain?

- Yes (1)
- No (2)

Skip To: End of Block If Q39.3 = No

| Page Break |  |
| --- | --- |

Q39.4 Please indicate the form(s) of percutaneous surgical therapy you have used in the past **12 months** for Parkinson's related pain. (Select all that apply)

- PENS (percutaneous electrical nerve stimulation) (1)
- PNT (percutaneous neuromodulation therapy) (2)
- Neurotomy (e.g. radiofrequency ablation, chemical ablation, rhizotomy) (3)
- Nerve blockade (e.g. injection of anaesthetic or steroid to a nerve) (4)
- Joint blockade (e.g. injection of anaesthetic or steroid to a joint) (5)
- Other percutaneous surgical therapy (please specify) (6) __________________________________________________

Q39.5 In the past **12 months**, how often have you used percutaneous surgical therapy for Parkinson's related pain?

- About once a month (1)
- Every few months (2)
- Once or twice a year (3)

Q39.6 In the past **12 months**, how effective, if at all, has percutaneous surgical therapy been in reducing your Parkinson's related pain?

- Not effective at all (1)
- Slightly effective (2)
- Moderately effective (3)
- Very effective (4)
- Extremely effective (5)

End of Block: PERCUTANEOUS SURGICAL THERAPY

Start of Block: SURGICAL THERAPY

Q40.1 **SUB-SECTION C.19 : SURGICAL THERAPY**

 This sub-section asks about **surgical therapy.**

 For this survey, the term ‘surgical therapy’ refers to surgical procedures typically performed by a neurosurgeon for the treatment of Parkinson’s and pain, such as **deep brain stimulation, spinal cord stimulation, unilateral pallidotomy, and MRI-guided focused ultrasound.**

 This does not include orthopaedic procedures such as total knee replacement surgery.

Q40.2 Have you ever had surgical therapy for the treatment of Parkinson's?

- Yes (1)
- No (2)

Skip To: End of Block If Q40.2 = No

| Page Break |  |
| --- | --- |

Q40.3 Please indicate the form(s) of surgical therapy you have had for the treatment of Parkinson's. (Select all that apply)

- Spinal cord stimulation (1)
- Deep brain stimulation (2)
- Unilateral pallidotomy (3)
- MRI-guided focused ultrasound (5)
- Other surgical therapy (please specify) (4) __________________________________________________

Q40.4 How effective, if at all, has surgical therapy been in reducing your Parkinson's related pain?

- Not effective at all (1)
- Slightly effective (2)
- Moderately effective (3)
- Very effective (4)
- Extremely effective (5)

End of Block: SURGICAL THERAPY

Start of Block: MIND-BODY THERAPY

Q41.1 **SUB-SECTION C.20 : MIND-BODY THERAPY**

 This sub-section asks about **mind-body therapy**.

 In this survey, the term ‘mind-body therapy’ refers to practices that involve mental focus, breathing, and body movements to help relax the body and mind such as **hypnosis, meditation, Tai Chi, biofeedback, and imagery**.

Q41.2 Have you ever used mind-body therapy for Parkinson's related pain?

- Yes (1)
- No (2)

Skip To: End of Block If Q41.2 = No

| Page Break |  |
| --- | --- |

Q41.3 In the past month, have you used mind-body therapy for Parkinson's related pain?

- Yes (1)
- No (2)

Skip To: End of Block If Q41.3 = No

| Page Break |  |
| --- | --- |

Q41.4 Please indicate the form(s) of mind-body therapy you have used in the past month for Parkinson's related pain. (Select all that apply)

- Hypnosis (1)
- Meditation (2)
- Tai Chi and Qi Gong (3)
- Biofeedback (4)
- Imagery (5)
- Other mind-body therapy (please specify) (6) __________________________________________________

Q41.5 In the past month, how often have you used mind-body therapy for Parkinson's related pain?

- Every day (9)
- Two to six times a week (10)
- Once a week (11)
- Less than once a week (12)

Q41.6 In the past month, how effective, if at all, has mind-body therapy been in reducing your Parkinson's related pain?

- Not effective at all (1)
- Slightly effective (2)
- Moderately effective (3)
- Very effective (4)
- Extremely effective (5)

End of Block: MIND-BODY THERAPY

Start of Block: ENERGY HEALING THERAPY

Q42.1 **SUB-SECTION C.21 : ENERGY HEALING THERAPY**

 This sub-section asks about energy healing therapy.

 In this survey, the term ‘energy healing therapy’ refers to practices that involve balancing energy flows through your body such as **reiki and therapeutic touch.**

Q42.2 Have you ever used energy healing therapy for Parkinson's related pain?

- Yes (1)
- No (2)

Skip To: End of Block If Q42.2 = No

| Page Break |  |
| --- | --- |

Q42.3 In the past month, have you used energy healing therapy for Parkinson's related pain?

- Yes (1)
- No (2)

Skip To: End of Block If Q42.3 = No

| Page Break |  |
| --- | --- |

Q42.4 Please indicate the form(s) of energy healing therapy you have used in the past month for Parkinson's related pain. (Select all that apply)

- Reiki (1)
- Therapeutic touch (2)
- Other energy healing therapy (please specify) (3) __________________________________________________

Q42.5 In the past month, how often have you used energy healing therapy for Parkinson's related pain?

- Every day (1)
- Two to six times a week (6)
- Once a week (7)
- Less than once a week (8)

Q42.6 In the past month, how effective, if at all, has energy healing therapy been in reducing your Parkinson's related pain?

- Not effective at all (1)
- Slightly effective (2)
- Moderately effective (3)
- Very effective (4)
- Extremely effective (5)

End of Block: ENERGY HEALING THERAPY

Start of Block: THERMOTHERAPY

Q43.1 **SUB-SECTION C.22 : THERMOTHERAPY**

 This sub-section asks about thermotherapy.

 In this survey, the term ‘thermotherapy’ refers to the application of **heat or cold** to parts of the body affected by pain.

Q43.2 Have you ever used thermotherapy for Parkinson's related pain?

- Yes (1)
- No (2)

Skip To: End of Block If Q43.2 = No

| Page Break |  |
| --- | --- |

Q43.3 In the past month, have you used thermotherapy for Parkinson's related pain?

- Yes (1)
- No (2)

Skip To: End of Block If Q43.3 = No

| Page Break |  |
| --- | --- |

Q43.4 Please indicate the form(s) of thermotherapy you have used in the past month for Parkinson's related pain. (Select all that apply)

- Cold compress/chemical cold pack/ice (1)
- Immersion or soaking in cold water (body part or whole body) (2)
- Hot compress/heat pads/hot water bottle (3)
- Immersion in warm/hot water (body part or whole body) (4)
- Heated paraffin wax (5)
- Heat rubs (e.g. Dencorub, Metsal, Tiger Balm) (6)
- Other thermotherapy (please specify) (7) __________________________________________________

Q43.5 In the past month, how often have you used thermotherapy for Parkinson's related pain?

- Every day (6)
- Two to six times a week (7)
- Once a week (8)
- Less than once a week (9)

Q43.6 In the past month, how effective, if at all, has thermotherapy been in reducing your Parkinson's related pain?

- Not effective at all (1)
- Slightly effective (2)
- Moderately effective (3)
- Very effective (4)
- Extremely effective (5)

End of Block: THERMOTHERAPY

Start of Block: ADDITIONAL THERAPIES

Q44.1 **SUB-SECTION C.23 : ADDITIONAL THERAPIES**

 In addition to the therapies mentioned in the previous sections, are there any other therapies that you have used for the treatment of Parkinson's related pain?

- Yes (1)
- No (2)

Skip To: End of Survey If Q44.1 = No

| Page Break |  |
| --- | --- |

Q44.2 What therapies have you used? (Please specify)

________________________________________________________________

End of Block: ADDITIONAL THERAPIES
